# Why do thioureas and squaramides slow down the Ireland–Claisen rearrangement?

**DOI:** 10.3762/bjoc.15.290

**Published:** 2019-12-10

**Authors:** Dominika Krištofíková, Juraj Filo, Mária Mečiarová, Radovan Šebesta

**Affiliations:** 1 Department of Organic Chemistry, Faculty of Natural Sciences, Comenius University in Bratislava, Mlynská dolina, Ilkovičova 6, 84215 Bratislava, Slovakia; 2 Institute of Chemistry, Faculty of Natural Sciences, Comenius University in Bratislava, Mlynská dolina, Ilkovičova 6, 84215 Bratislava, Slovakia

**Keywords:** DFT calculations, green solvents, H-bonding catalysts, Ireland–Claisen rearrangement, silyl ketene acetals

## Abstract

A range of chiral hydrogen-bond-donating organocatalysts was tested in the Ireland–Claisen rearrangement of silyl ketene acetals. None of these organocatalysts was able to impart any enantioselectivity on the rearrangements. Furthermore, these organocatalysts slowed down the Ireland–Claisen rearrangement in comparison to an uncatalyzed reaction. The catalyst-free reaction proceeded well in green solvents or without any solvent. DFT calculations showed that the activation barriers are higher for reactions involving hydrogen-donating organocatalysts and kinetic experiments suggest that the catalysts bind stronger to the starting silyl ketene acetals than to transition structures thus leading to inefficient rearrangement reactions.

## Introduction

The Ireland–Claisen rearrangement is a reaction converting allyl esters to γ,δ-unsaturated carboxylic acids. Its key step is a [3,3]-sigmatropic rearrangement of a silyl ketene acetal, which is generated in situ by deprotonation of an allyl ester using a strong base [1–3]. The products of the Ireland–Claisen rearrangement, γ,δ-unsaturated acids, are useful precursors of biologically active compounds and natural products [4–11]. The ready availability of allylic esters, the ability to control the *E*/*Z* geometry of enolates as well as its stereospecificity make this transformation synthetically appealing [12–13].

Allyl esters of various carboxylic acids undergo rearrangement as their lithium enolates or silyl ketene acetals and the corresponding acids were isolated in 75–80% yields. Accordingly, the Ireland–Claisen rearrangement of lithium enolates generated from allyl fluoroacetates gave the corresponding α-fluoro-γ,δ-unsaturated acids [14]. The Ireland–Claisen rearrangement of Et_3_N-solvated enolates showed higher reactivity as well as diastereoselectivity when compared with analogous reactions in THF [15].

The required ester enolates are typically generated using a strong base, such as LDA or similar amides in combination with trialkylsilyl chlorides as silylating agents at low temperature under strictly anhydrous conditions [3,16–17]. An alternative method employs tertiary amines as bases in combination with more reactive silylating agents, such as trialkylsilyl triflates [18–19].

The rearrangement of ester enolates generated by LDA with metal ions bearing bulky cyclopentadienyl ligands proceeded well with yields of up to 90%, and diastereomeric ratios strongly depended on the ligands used [20]. The presence of catalytic amounts of Lewis acids improved the diastereoselectivity and the reaction rate of silyl ketene acetals of (*E*)-allylic esters [21]. Chiral bromoboranes were used to form boranyl ketene acetals from ester enolates generated from allyl esters with tertiary amines. The geometry of the enolates depended strongly on the solvent and the amine’s structure [22–23].

The decomposition of the enolate is one of the side reactions, which can be suppressed by chelation. The chelation-stabilized enolates are more stable than the corresponding silyl ketene acetals, and they are capable of a direct rearrangement [24]. The chelation-controlled Ireland–Claisen rearrangement of *O*-protected allylic glycolate esters proceeded with moderate yields and diastereoselectivities of up to 20:1 [25]. An asymmetric ester-enolate-Claisen rearrangement was achieved by using aluminum-chelate-bridged enolates and proceeded with high yields and diastereoselectivities, and ees up to 86% [26]. Rearrangement of allyl esters of glycine derivatives gave under similar conditions amino acids with a quaternary stereocenter on the β-carbon with high yields and excellent diastereo- as well as enantioselectivity [5]. A reductive rearrangement of allyl esters of acrylic acid, catalyzed by in situ-generated copper hydride with diethoxymethylsilane as a reductive agent gave the γ,δ-unsaturated acid with good to excellent diastereoselectivities [27].

Various asymmetric organocatalyzed rearrangements are known [28]. However, the catalysis of sigmatropic rearrangements is difficult due to their rather nonpolar transition states, which are difficult to be addressed by catalysts [29]. Several stereoselective [3,3]-sigmatropic rearrangements are realized with chiral Brønsted acids [30–34]. Jacobsen reported guanidinium-catalyzed enantioselective Claisen rearrangements of *O*-allyl β-ketoesters [35–37]. Hiersemann and Strassner studied the Claisen rearrangement with H-bond-donating organocatalysts by computational methods and concluded that thioureas are not efficient in transition-state stabilization [38].

Regarding the usefulness of the Ireland–Claisen rearrangement, we tried to optimize its reaction course under mild conditions using various bases, solvents, and hydrogen-bond-donating catalysts. We also present a computational explanation and NMR kinetic study for the inefficient Ireland–Claisen rearrangement under thiourea and squaramide catalysis.

## Results and Discussion

**Scheme 1:**
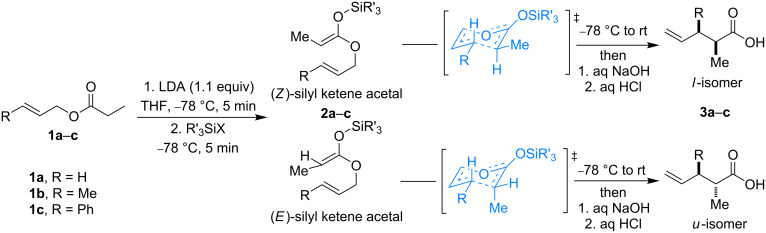
Ireland–Claisen rearrangement of allyl esters **1a**–**c**.

We started our investigation with the rearrangement of trimethylsilyl ketene acetal (**2a**) derived from allyl propionate (**1a**, Scheme 1). Silyl ketene acetals **2** can be observed by NMR in the reaction mixture (see Supporting Information File 1 for NMR spectra of **2c**). This reaction afforded the corresponding acid **3a** in 63% yield at rt (Table 1, entry 1). At a higher temperature, the product yield decreased (Table 1, entry 2). The reaction of the related (*E*)-but-2-en-1-yl propionate (**1b**) gave acid **3b** under similar conditions with slightly lower yields. The rearrangement of lithium enolate, as well as trimethylsilyl ketene acetal **2b** generated from **1b**, proceeded with similar yields and diastereoselectivity (Table 1, entries 3–6). However, the Ireland–Claisen rearrangement attempted with cinnamyl propionate (**1c**) did not take place when using LDA as a base (Table 1, entries 7 and 8).

**Table 1:** Ireland–Claisen rearrangement of **1a**–**c** with LDA as the base without organocatalysts.

Entry	R	R’_3_SiX (equiv)	Temperature (°C)	Time (h)	Yield of **2** (%)	dr

1	H	Me_3_SiCl (1.1)	rt	168	63	–
2	H	Me_3_SiCl (1.1)	66	3	41	–
3	Me	Me_3_SiCl (1.1)	rt	72	42	76:24
4	Me	Me_3_SiCl (1.1)	66	2	36	76:24
5	Me	Me_3_SiCl (1.1)	rt	20	38	77:23
6	Me	–	rt	20	30	79:21
7	Ph	Me_3_SiCl (1.0)	rt	168	0	–
8	Ph	*t-*BuMe_2_SiOTf (1.1)	rt	168	0	–

**Scheme 2:**
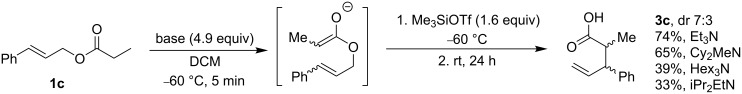
Ireland–Claisen rearrangement of **1c** mediated by tertiary amines.

Next, we studied the Ireland–Claisen rearrangement of ester **1c** using various tertiary amines as bases. The ester enolate was trapped with Me_3_SiOTf at −60 °C and allowing the reaction to proceed at room temperature for 24 h. The best yields of acid **3c** were achieved with triethylamine (74%) and *N*-cyclohexyl-*N*-methylcyclohexanamine (65%, Scheme 2) whereas reactions with trihexylamine and diisopropylethylamine provided the product **3c** with lower yields. Rearrangement in the presence of *N*-methylmorpholine, quinine, and *N*-methylpyrrolidine afforded only traces of acid **3c** and reactions with DBU, DABCO and (*S*)-1-(pyrrolidin-2-ylmethyl)pyrrolidine did not proceed at all. Of note, the bases used in these Ireland–Claisen rearrangements did not affect the diastereoselectivity and the *syn*/*anti* ratio was approximately 70:30 in all cases.

The Ireland–Claisen rearrangement of allyl propionate (**1a**) with Et_3_N and Me_3_SiOTf afforded 2-methylpent-4-enoic acid (**3a**) in 75% yield. However, the yield of product **3a** dropped to 22% when the reaction was performed at 40 °C. The rearrangement of **1a** with *t*-BuMe_2_SiOTf gave only 40% of acid **3a** (Table 2, entries 1–3). The yield of acid **3c** decreased from 74 to 50% when the reaction was performed at 5 °C (Table 2, cf. entries 4 and 5) and the reaction temperature did not affect the diastereoselectivity of the reaction. No product **3c** was obtained when the reaction was performed without silyl triflate (Table 2, entry 6).

**Table 2:** Ireland–Claisen rearrangement with Et_3_N as a base.

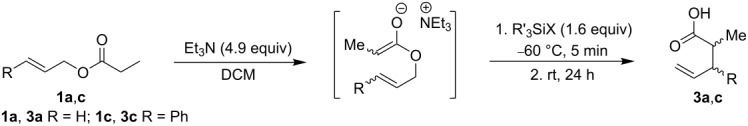						

Entry	R	R’_3_SiOTf	*T* (°C)	*t* (h)	Yield of **3** (%)	dr

1	H	Me_3_SiOTf	rt	24	75	–
2	H	Me_3_SiOTf	40	4	22	–
3	H	*t*-BuMe_2_SiOTf	rt	24	40	–
4	Ph	Me_3_SiOTf	rt	24	74	69:31
5	Ph	Me_3_SiOTf	5	72	50	71:29
6	Ph	–	rt	24	0	–

**Figure 1:**
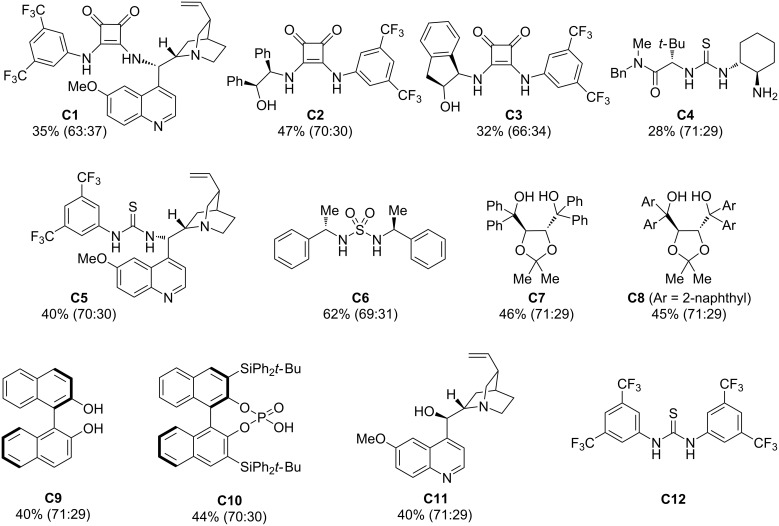
Organocatalysts used in this study. Conditions: typical procedure: 1. Et_3_N (4.9 equiv), DCM, −60 °C, 5 min; 2. Me_3_SiOTf (1.6 equiv), catalyst (10 mol %), −60 °C; 3. rt, 24 h.

We assumed that hydrogen-bond-donating organocatalysts could activate the enolates or the corresponding silyl ketene acetals or stabilize the corresponding transition states. In addition, chiral organocatalysts could induce diastereo- as well as enantioselectivity. Therefore, we examined the Ireland–Claisen rearrangement of ester **1c** in the presence of a range of organocatalysts, including chiral squaramides, thioureas, alkaloids and their derivatives, taddols, binol, chiral phosphoric acid, (*S*)-proline and its derivatives, amide of tryptophan (Figure 1). These catalysts feature varying steric properties from sterically encumbered, such as **C1**, **C5**, **C8**, or **C10**, to less demanding ones, e.g., **C6**, **C9** or **C11**. In addition, the acidity of the hydrogen-bond-donating moiety also ranges over a rather large area from p*K*a (H_2_O) 1 for phosphoric acid **C10** to p*K*a (DMSO) 28 of diols **C7** and **C8**. However, neither steric factors, nor the acidity of the H-bond-donor moiety seemed to play a significant role in the Ireland–Claisen rearrangement of **1c**. Surprisingly, all catalysts led to a decreased yield of the acid **3c** (28–62%) in comparison to 74% yield obtained without any chiral organocatalyst. Further, none of the chiral organocatalysts considerably affected the diastereoselectivity and acid **3c** was obtained in racemic form in the presence of either tested chiral organocatalyst. Similar results were obtained with ester **1a**. The reaction was tested also in the more polar solvent acetonitrile using catalyst **C1**, but also in this case no enantioselectivity was observed. Sigmatropic rearrangements proceed via isopolar transition states and, therefore solvent effects are rather small. For this reason and due to the complete lack of enantioselectivity, at this stage, we did not investigate other solvents with the catalysts collected in Figure 1.

In order to gain further insight into the catalyst’s action in the rearrangement, we have studied the effect of catalyst loading in the reaction of ester **1c** with squaramide **C1**. The reaction with Et_3_N as a base and Me_3_SiOTf without any chiral organocatalyst afforded acid **3c** with 74% yield. Repeating the reaction in the presence of squaramide **C1** (5 mol %) lowered the yield from 74 to 37%. A similar yield (35%) was achieved with 10 mol % of **C1** (Table 3, entries 1–3) and further increasing the catalyst loading to 20 and 30 mol % diminished the product yield even more (26 and 21%, respectively; Table 3, entries 4 and 5). Ultimately, one equivalent of squaramide **C1** completely stopped the reaction (Table 3, entry 6).

**Table 3:** Effect of catalyst loading on the Ireland–Claisen rearrangement of **1c**.

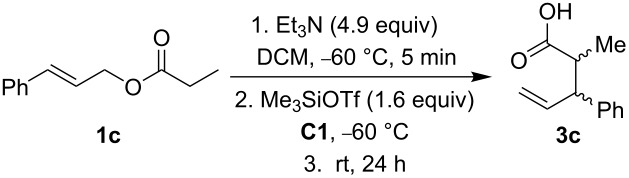			

Entry	**C1** (mol %)	Yield of **3c** (%)	*syn*/*anti*

1	0	74	69:31
2	5	37	71:29
3	10	35	63:37
4	20	26	74:26
5	30	21	75:25
6	100	0	–

We also studied the effect of the amount of base on the Ireland–Claisen rearrangement of ester **1c** in the presence of sulfanediamine **C6** (Table 4). The best yield of acid **3c** (74%) was recorded with 4.9 equivalents of Et_3_N without chiral organocatalyst. The yield of acid **3c** decreased to 62% in the presence of 10 mol % of **C6**. An even higher amount of base did not improve the yield of acid **3c** (Table 4, entry 3). The reaction with 2.5 equivalents of Et_3_N gave 51% of acid **3c**. Also here, both diastereomers were obtained as racemates.

**Table 4:** Effect of the amount of base on the Ireland–Claisen rearrangement of **1c** in the presence of **C6**.

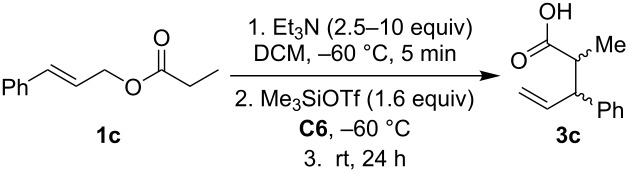				

Entry	Et_3_N (equiv)	**C6** (mol %)	Yield of **3c** (%)	*syn*/*anti*

1	4.9	0	74	69:31
2	4.9	10	62	69:31
3	10	10	70	71:29
4	2.5	10	51	64:36

As mentioned above, the Ireland–Claisen rearrangement, as a pericyclic reaction, is expected to be rather insensitive to solvent effects. However, minor improvements in reaction time have been noted in some cases [39]. Therefore, with the aim of improving the reaction course, we evaluated several other solvents, emphasizing green solvents, which have not been evaluated in Ireland–Claisen rearrangements before. 2-Methyltetrahydrofuran (2-MeTHF) is derived from renewable resources and has a higher boiling point (80 °C) and lower heat of vaporization compared to THF. The Ireland–Claisen rearrangement proceeded in 2-MeTHF with slightly lower yield and very similar diastereoselectivity as in DCM (Table 5, entries 1 and 2). A low tendency to peroxide formation, stability under acidic and basic conditions, high boiling point, and low heat of vaporization are also positive features of cyclopentyl methyl ether (CpOMe) [40]. From the chemical yield point of view, cyclopentyl methyl ether was the best solvent for the Ireland–Claisen rearrangement of ester **1c**; acid **3c** was isolated in 84% yield. However, even in CpOMe, catalyst **C6** decreased the yield of acid **3c** to 73% (Table 5, entries 3 and 4). A less toxic alternative to dichloromethane is trifluorotoluene and the rearrangement of **1c** in this solvent afforded acid **3c** in 76% (Table 5, entry 5). Glymes are environmentally benign aprotic polar and chemically inert solvents [41]. The desired product **3c** through Ireland–Claisen rearrangement of **1c** was isolated in 58% when the reaction was carried out in dimethyl ethylene glycol (Table 5, entry 6). However, Ireland–Claisen rearrangement of ester **1c** did not proceed in 2,2,2-trifluoroethanol, ethyl ʟ-lactate or in 2-butanone.

Interestingly, the best yield of acid **3c** (95%) was obtained when the reaction was carried out without any solvent (Table 5, entry 8). Again, catalyst **C6** (10 mol %) decreased the yield under solvent-free conditions from 95 to 73% (Table 5, entries 8 vs 9).

**Table 5:** Green solvents screening for the Ireland–Claisen rearrangement of **1c**.

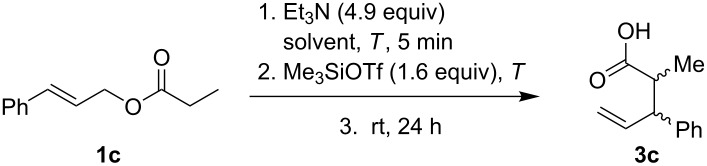				

Entry	Solvent	*T* (°C)	Yield of **3c** (%)	*syn*/*anti^a^*

1	DCM	−60	74	69:31
2	2-Me-THF	−60	63	71:29
3	CpOMe	−60	84	68:32
4	CpOMe^b^	−60	73	70:30
5	PhCF_3_	−20	76	69:31
6	CH_3_OCH_2_CH_2_OCH_3_	−50	58	70:30
7	CH_3_CN	−60	11	n.d.
8	–	−60	95	69:31
9	–^b^	−60	73	70:30

^a^Both diastereomers were obtained as a racemic mixture. ^b^Reaction carried out in the presence of **C6** (10 mol %).

**Scheme 3:**
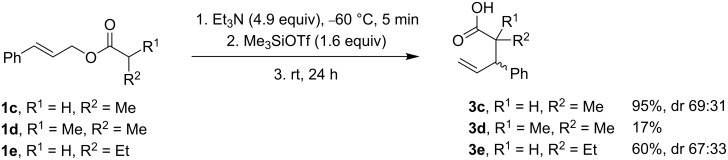
Solvent-free Ireland–Claisen rearrangement of cinnamyl esters.

The Ireland–Claisen rearrangement of cinnamyl isobutyrate (**1d**) gave under solvent-free conditions only 17% of the acid **3d** and the analogous reaction in CpOMe afforded the acid in 5% yield. When cinnamyl butyrate (**1e**) was used as a starting material, product **3e** was obtained in a yield of 60% (Scheme 3).

**Figure 2:**
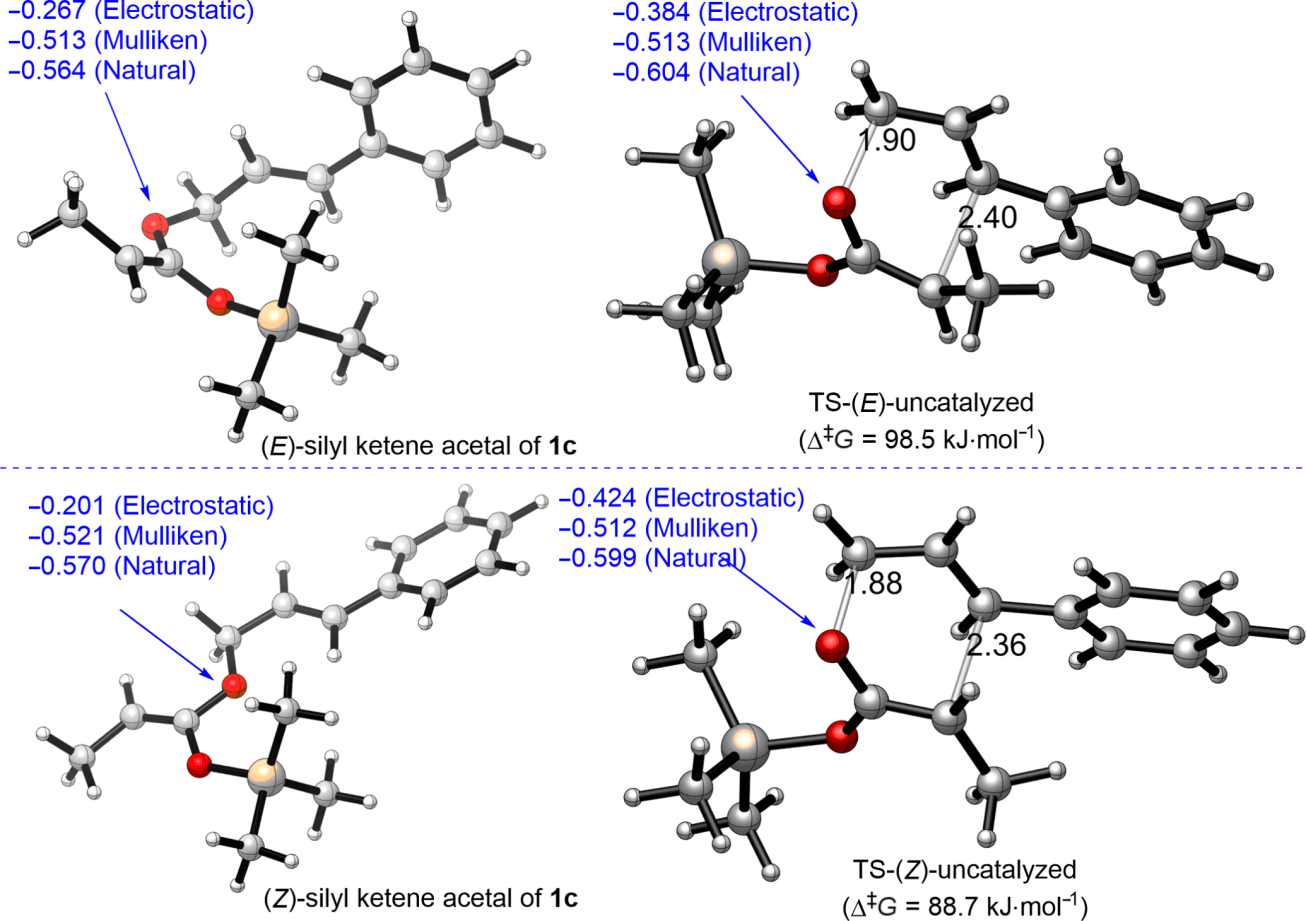
ωB97X-D/6-31G* calculated uncatalyzed Ireland–Claisen rearrangement of **1c**. Charges on allylic oxygen (blue); C–O bond-breaking and C–C bond-forming distances in TS (black).

To gain further insights into the organocatalysts effect on the Ireland–Claisen rearrangement, we have performed quantum-chemical calculations employing long-range corrected hybrid density ωB97X-D functional [42]. This dispersion-corrected functional displays very balanced overall performances and has demonstrated excellent treatment of noncovalent interactions [43], which are very important in our studied system. For this, we have compared the uncatalyzed Ireland–Claisen rearrangement with three hydrogen-bond-catalyzed reactions. As model H-bonding catalysts, we selected diphenylthiourea, Schreiner thiourea, and the corresponding squaramide with bis(trifluoromethyl)phenyl groups. We have evaluated both the (*E*)- and (*Z*)-silyl ketene acetal **2c** derived from **1c** as starting material for the reaction. The activation barrier for the uncatalyzed reaction of (*E*)-silyl ketene acetal was 98.5 kJ·mol^−1^, and for (*Z*)-silyl ketene acetal 88.7 kJ·mol^−1^ (Figure 2). Charges on the allylic oxygen in the reaction transition states are only slightly more negative than in the starting acetal silyl ketenes. It means that transition states have only slightly dipolar character, which would be difficult to stabilize through hydrogen bonding and consequently reaction less prone to catalysis.

**Figure 3:**
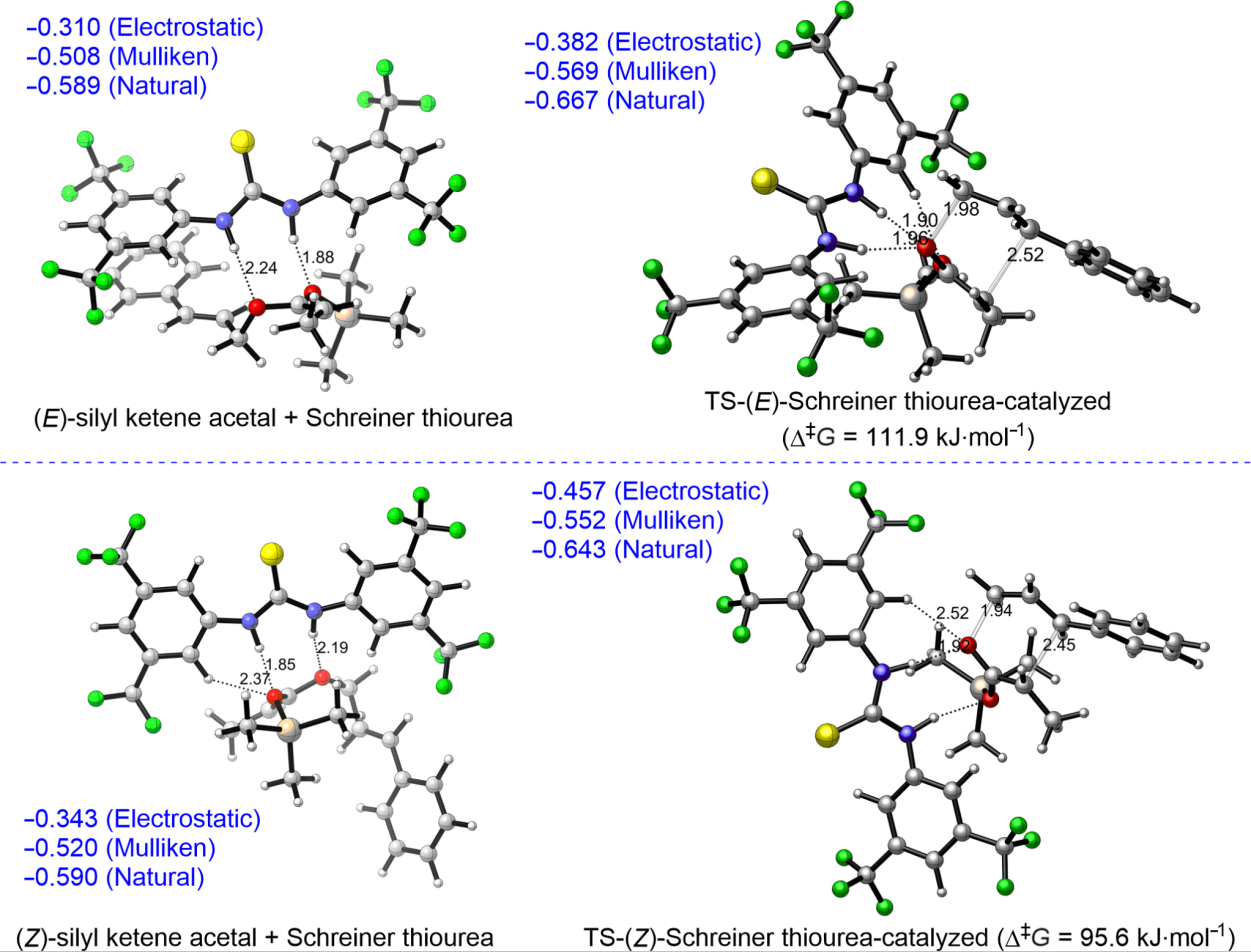
ωB97X-D/6-31G* calculated Schreiner thiourea (**12**)-catalyzed Ireland–Claisen rearrangement of **1c**. Charges on allylic oxygen (blue); C–O bond-breaking and C–C bond-forming distances in TS (black); hydrogen-bond distances (black).

Interestingly, the reaction comprising Schreiner thiourea (**C12**) had higher activation barriers for both isomers (111.9 kJ·mol^−1^ for (*E*) and 95.6 kJ·mol^−1^ for (*Z*)). These results suggest that Schreiner thiourea binds stronger to starting silyl ketene acetal than to the corresponding transition state. Therefore, it stabilizes more the starting material than the transition state, which results in the slow-down of the reaction (Figure 3).

**Figure 4:**
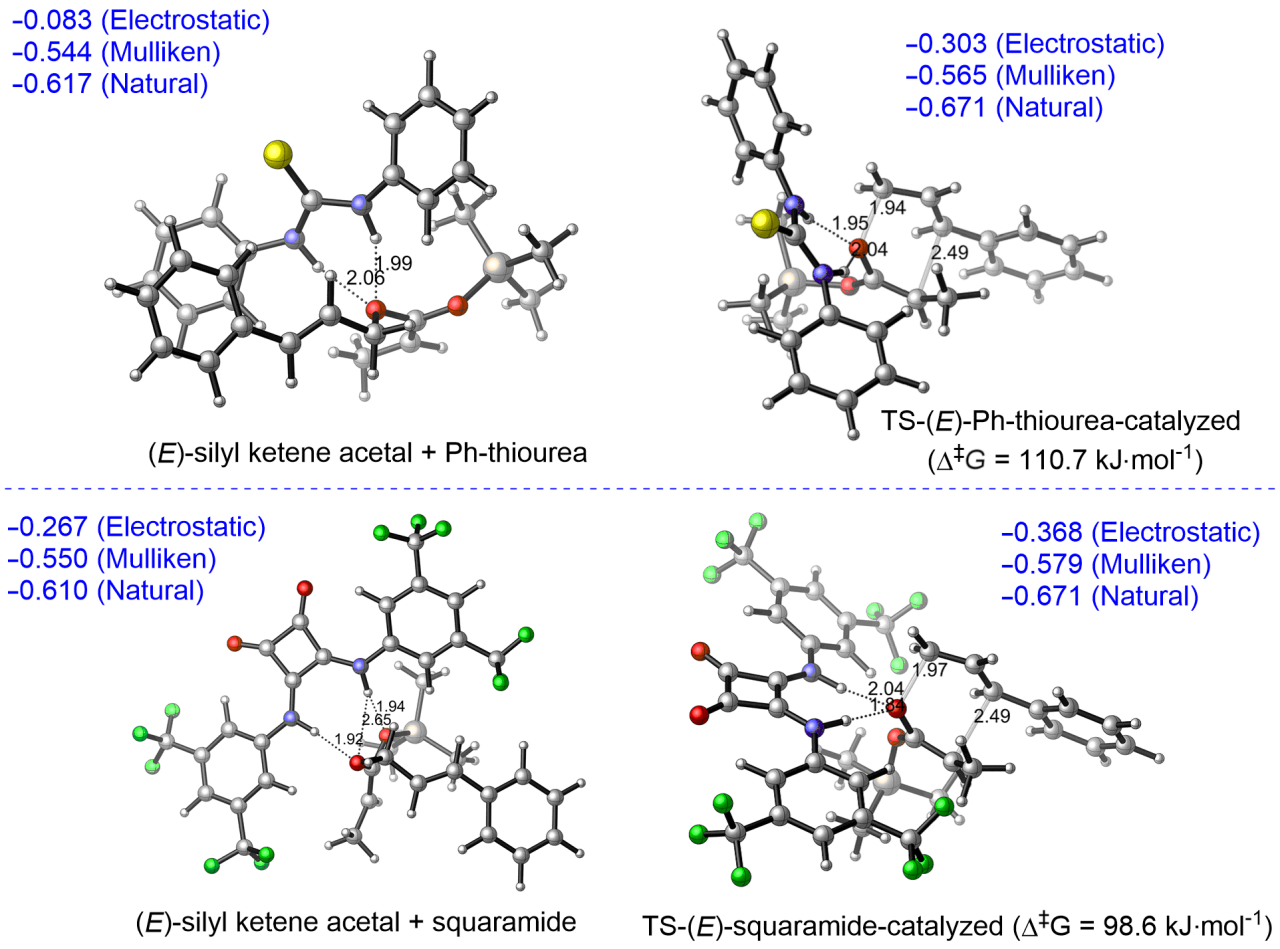
ωB97X-D/6-31G* calculated Ph-thiourea (top) and squaramide-catalyzed (bottom) Ireland–Claisen rearrangement of **1c**. Charges on allylic oxygen (blue); C–O bond-breaking and C–C bond-forming distances in TS (black); hydrogen-bond distances (black).

Similar trends were observed also for diphenylthiourea and a squaramide (Figure 4).

Using the structures optimized at the ωB97X-D/6-31G* level of theory we calculated interaction energies between catalysts and starting silyl ketene acetals or corresponding transition state structures at the M06-2X/6-311+G** level. For the Schreiner thiourea (**C12**) as well as diphenylthiourea interaction energies were higher in the starting silyl ketene acetal–catalyst complexes than in the corresponding transition states. For the squaramide catalyst, these energies were similar (Table 6). These calculations further support the notion that hydrogen-bonding organocatalysts bind stronger to starting silyl ketene acetals than to the transition structure and thus are not efficiently catalyzing the Ireland–Claisen rearrangement. The reason for this difference, however, remains unclear. The explanation may be connected to a more compact transition state of sigmatropic rearrangements than their starting materials, which is thus less amenable to additional stabilization via hydrogen-bonding catalysts.

**Table 6:** Comparison of DFT-calculated interaction energies between starting silyl ketene acetal/transition state structure and catalysts.

Structure	Δ*E* (kJ·mol^−1^)^a^	Structure	Δ*E* (kJ·mol^−1^)^a^

*(E)*-**2c** (**C12**)	−135.5	(*E*)-**2c** (Ph-thiourea)	−91.7
TS-*(E)*-**2c** (**C12**)	−105.0	TS-(*E*)-**2c** (Ph-thiourea)	−61.8
(*Z*)-**2c** (**C12**)	−112.1	(*E*)-**2c** (squaramide)	−119.2
TS-(*Z*)-**2c** (**C12**)	−98.0	TS-(*E*)-**2c** (squaramide)	−122.7

^a^Calculated at ωB97X-D/6-31G*// M06-2X/6-311+G** level as a difference between electronic energy of **2c**–catalyst (or TS-**2c**–catalyst) complex and sum of individual components.

**Figure 5:**
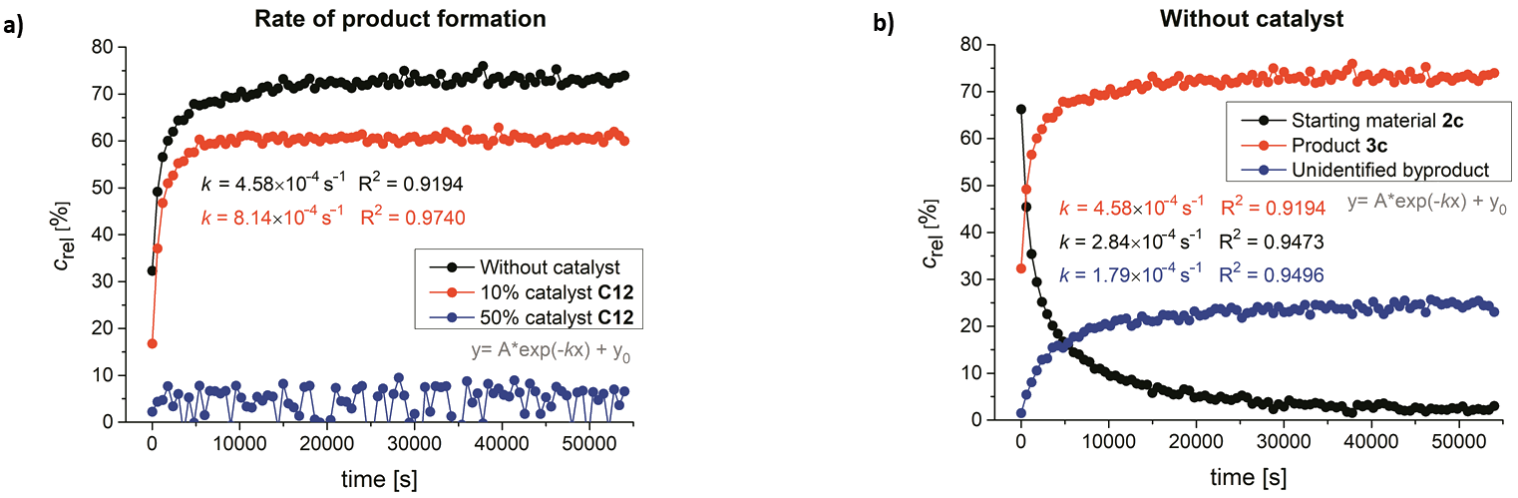
a) Rate of product formation; b) reaction profile without catalyst determined by ^1^H NMR.

To get further insight, we have performed a kinetic study of the reaction using ^1^H NMR spectroscopy. Figure 5a shows a comparison of product formation in the presence of 0, 10, and 50 mol % of catalyst **C12**. As can be seen, the catalyst hindered product formation and at 50 mol % catalyst loading product **3c** practically does not form. A reaction profile without catalyst is displayed in Figure 5b. In this case, the formation of a small amount of an unidentified byproduct was also observed. The rate constants for the uncatalyzed and catalyzed reaction are similar. The activation Gibbs energy was determined to be approximately 90 kJ·mol^−1^, which agrees well with the DFT calculated values. These kinetic measurements support the notion that H-bonding catalysts bind stronger to starting silyl ketene acetals and thus prevent it from undergoing Ireland–Claisen rearrangement. For more details, see Supporting Information File 1.

## Conclusion

Chiral hydrogen-bond-donating organocatalysts such as thioureas, squaramides, or alcohols failed to catalyze the Ireland–Claisen rearrangement of silyl ketene acetals. Comparison experiments showed that increasing catalyst loading gradually slowed-down the reaction. DFT calculations using long-range corrected hybrid density ωB97X-D functional showed that thioureas and squaramides stabilize the starting ground state more than the corresponding transitions states. This fact leads to a higher activation barrier and slower reactions in the presence of hydrogen-bond donating organocatalysts. On the other hand, catalyst-free Ireland–Claisen rearrangements proceed well in green solvents such as CpOMe or under solvent-free conditions. NMR kinetic measurements supported the notion that thiourea and squaramide organocatalysts bind strongly to the starting silyl ketene acetals and prevent the Ireland–Claisen rearrangement. Further experiments towards finding more effective organocatalysts are underway in our laboratory.

## Experimental

### A typical procedure for Ireland–Claisen rearrangement with Et_3_N

Ester **1** (2.34 mmol) was added to a solution of Et_3_N (1.6 mL, 11.5 mmol) in dry CH_2_Cl_2_ (2.2 mL) under a nitrogen atmosphere and the reaction mixture was cooled to −60 °C. Then, trialkyl silyl triflate (3.7 mmol) was added dropwise, followed by addition of the organocatalyst (10 mol %) in one portion. The reaction temperature was allowed to reach ambient temperature and the reaction mixture was stirred at this temperature for 24 h. Afterwards, the solvent was evaporated under reduced pressure and Et_2_O (5 mL) and 1.3 M NaOH (7 mL) were added to the residue. The aqueous layer was washed with CH_2_Cl_2_ (3 × 8 mL), and then it was acidified with concentrated HCl. The products were extracted with CH_2_Cl_2_ (3 × 8 mL). The organic solution was washed with water (3 × 12 mL) and dried with anhydrous MgSO_4_. The solvent was evaporated under reduced pressure.

## Supporting Information

File 1 Experimental procedures and characterization data for all compounds, copies of NMR spectra, details of DFT calculations.

## References

[1] Ireland R E, Mueller R H (1972). J Am Chem Soc.

[2] Ireland R E, Willard A K (1975). Tetrahedron Lett.

[3] Ireland R E, Wilcox C S (1977). Tetrahedron Lett.

[4] Davies S G, Fletcher A M, Roberts P M, Thomson J E, Zammit C M (2013). Chem Commun.

[5] Krebs A, Kazmaier U (1996). Tetrahedron Lett.

[6] Srikrishna A, Khan I A, Babu R R, Sajjanshetty A (2007). Tetrahedron.

[7] Churcher I, Williams S, Kerrad S, Harrison T, Castro J L, Shearman M S, Lewis H D, Clarke E E, Wrigley J D J, Beher D (2003). J Med Chem.

[8] Kraft P, Denizot N (2013). Eur J Org Chem.

[9] Liu D, Yu X, Huang L (2013). Chin J Chem.

[10] Dittrich N, Jung E-K, Davidson S J, Barker D (2016). Tetrahedron.

[11] Tellam J P, Carbery D R (2010). J Org Chem.

[12] Ito H, Taguchi T (1999). Chem Soc Rev.

[13] Chai Y, Hong S-p, Lindsay H A, McFarland C, McIntosh M C (2002). Tetrahedron.

[14] Welch J T, Samartino J S (1985). J Org Chem.

[15] Godenschwager P F, Collum D B (2008). J Am Chem Soc.

[16] Welch J T, Plummer J S, Chou T S (1991). J Org Chem.

[17] Liu D, Yu X (2012). Tetrahedron Lett.

[18] Kobayashi M, Masumoto K, Nakai E-i, Nakai T (1996). Tetrahedron Lett.

[19] Araki K, Welch J T (1993). Tetrahedron Lett.

[20] Uchiyama H, Kawano M, Katsuki T, Yamaguchi M (1987). Chem Lett.

[21] Koch G, Janser P, Kottirsch G, Romero-Giron E (2002). Tetrahedron Lett.

[22] Corey E J, Lee D H (1991). J Am Chem Soc.

[23] Seizert C A, Ferreira E M (2014). Chem – Eur J.

[24] Kazmaier U (1997). Liebigs Ann/Recl.

[25] Burke S D, Fobare W F, Pacofsky G J (1983). J Org Chem.

[26] Kazmaier U, Krebs A (1995). Angew Chem, Int Ed Engl.

[27] Wong K C, Ng E, Wong W-T, Chiu P (2016). Chem – Eur J.

[28] Moyano A, El-Hamdouni N, Atlamsani A (2010). Chem – Eur J.

[29] Tantillo D J (2016). Acc Chem Res.

[30] Li G-Q, Gao H, Keene C, Devonas M, Ess D H, Kürti L (2013). J Am Chem Soc.

[31] Wang J-Z, Zhou J, Xu C, Sun H, Kürti L, Xu Q-L (2016). J Am Chem Soc.

[32] Gao H, Xu Q-L, Keene C, Yousufuddin M, Ess D H, Kürti L (2016). Angew Chem, Int Ed.

[33] Rueping M, Antonchick A P (2008). Angew Chem, Int Ed.

[34] Maity P, Pemberton R P, Tantillo D J, Tambar U K (2013). J Am Chem Soc.

[35] Uyeda C, Jacobsen E N (2008). J Am Chem Soc.

[36] Uyeda C, Rötheli A R, Jacobsen E N (2010). Angew Chem, Int Ed.

[37] Uyeda C, Jacobsen E N (2011). J Am Chem Soc.

[38] Kirsten M, Rehbein J, Hiersemann M, Strassner T (2007). J Org Chem.

[39] Ganem B (1996). Angew Chem, Int Ed Engl.

[40] Shanab K, Neudorfer C, Spreitzer H (2016). Curr Org Chem.

[41] Tang S, Zhao H (2014). RSC Adv.

[42] Chai J-D, Head-Gordon M (2008). Phys Chem Chem Phys.

[43] Mardirossian N, Head-Gordon M (2017). Mol Phys.

