# Supplementary material for: Why do thioureas and squaramides slow down the Ireland–Claisen rearrangement?
Source: Beilstein J Org Chem. 2019 Dec 10;15:2948–57. doi: 10.3762/bjoc.15.290 (PMC6941421; doi:10.3762/bjoc.15.290)
Supplement: File 1 — Experimental procedures and characterization data for all compounds, copies of NMR spectra, details of DFT calculations. [file Beilstein_J_Org_Chem-15-2948-s001.pdf]

BEILSTEIN JOURNAL OF ORGANIC CHEMISTRY

Supporting Information

for

Why do thioureas and squaramides slow down the Ireland–Claisen rearrangement?

Dominika Krištofíková, Juraj Filo, Mária Mečiarová and Radovan Šebesta

Beilstein J. Org. Chem. **2019,** *15,* 2948–2957. doi:10.3762/bjoc.15.290

Experimental procedures and characterization data for all compounds, copies of NMR spectra, details of DFT calculations

License and Terms: This is a supporting information file under the terms of the Creative Commons Attribution License (http://creativecommons.org/licenses/by/4.0). Please note that the reuse, redistribution and reproduction in particular requires that the authors and source are credited.
The license is subject to the *Beilstein Journal of Organic Chemistry* terms and conditions: (https://www.beilstein-journals.org/bjoc)

Table of contents

pKa Values for catalysts S1

Materials and methods S2

Typical procedure for Ireland–Claisen rearrangement with Et_3N as a base S2

Synthesis of methyl esters for HPLC analysis S2

Typical procedure for Ireland–Claisen rearrangement with LDA as a base S3

Characterization data S3

Computational details S10

Kinetic measurements S28

p*K*a Values for catalysts

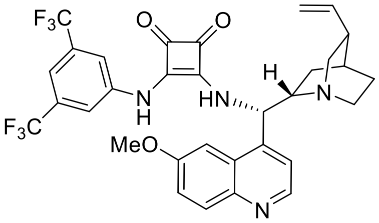

C1
pKa(DMSO) 15.0
(*Molecules* **2015**, *20*, 15500)

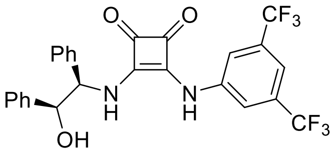

C2
pKa(DMSO) 8.3-16.5
(*Org. Lett.* **2014**, *16*, 1786)

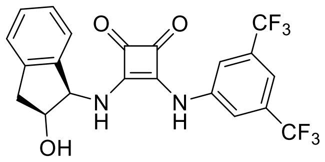

C3
pKa(DMSO) 8.3-16.5
(*Org. Lett.* **2014**, *16*, 1786)

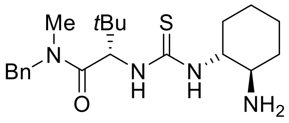

C4
pKa(DMSO) 19.5-21.0
(*Molecules* **2015**, *20*, 15500)

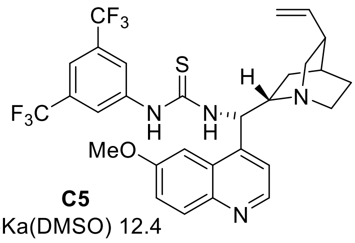

C5
pKa(DMSO) 12.4
(*Org. Lett.* **2014**, *16*, 1786)

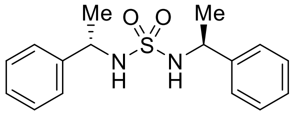

C6
pKa(DMSO) 16.0-17.5
(*J. Org. Chem.* **1990**, *55*, 3330)

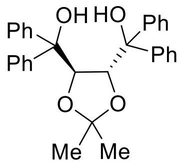

C7
pKa(DMSO) 28.0
(*Chem. Rev.* **2007**, *107*, 5713)

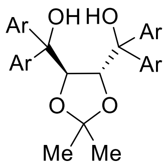

C8 (Ar = 2-Naphthyl)
pKa(DMSO) 28.0
(*Chem. Rev.* **2007**, *107*, 5713)

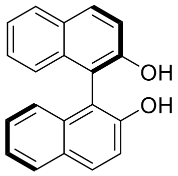

C9
pKa(DMSO) 13.0
(*Org. Chem. Front.* **2016**, *3*, 1154)

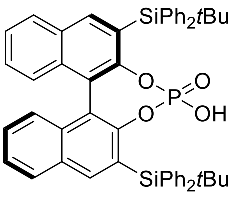

C10
pKa(H_2O) 1.0
(*Chem. Rev.* **2007**, *107*, 5713)

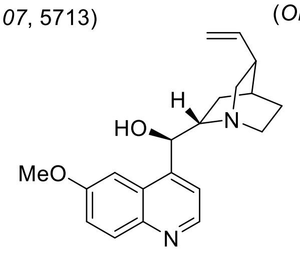

C11

Materials and methods

All commercially available chemicals were purchased from Sigma-Aldrich, Alfa Aesar or Merck and were used without further purification. Solvents were distilled and dried according to the standard procedures. All reactions were carried out in an inert atmosphere. Thin-layer chromatography was performed on silica gel 60, F-254 nm plates. Compounds were visualized with UV light (254 nm) and/or by treatment with $KMnO_4$ and anisaldehyde solution. NMR spectra were recorded on Varian NMR System 300 (300 or 600 MHz for ^{1}H and 75 or 151 MHz for ^{13}C). Chemical shifts (δ) are given in ppm relative to tetramethylsilane. Enantiomeric purity was determined by chiral HPLC column (OJ-H).

Typical procedure for Ireland–Claisen rearrangement with Et_3N as a base[1]

Ester **1** (2.34 mmol) was added into solution of Et_3N (1.6 mL, 11.51 mmol) in dry CH_2Cl_2 (2.2 mL) under a nitrogen atmosphere. The reaction mixture was cooled to −60 °C. Then, trialkyl silyl triflate (3.7 mmol) was added dropwise. Next, an organocatalyst (10 mol %) was added in one portion. The reaction temperature was allowed to reach ambient temperature and the reaction mixture was stirred at this temperature for 24 h. After the reaction, the solvent was evaporated under reduced pressure. Diethyl ether (5 mL) and 1.3 M NaOH (7 mL) were added to the distillation residue. The aqueous layer was washed with CH_2Cl_2 (3 × 8 mL) and after that it was acidified with concentrated HCl. The products were extracted with CH_2Cl_2 (3 × 8 mL). The organic phase was washed with water (3 × 12 mL), dried with anhydrous $MgSO_4$ and the solvent was evaporated under reduced pressure.

Synthesis of methyl esters for HPLC analysis [2]

A solution of diazald (1 g) in a mixture of 1,2-dimethoxyethane/diethyl ether 1:1 (3 mL) and a solution of KOH (1 g) in a mixture of water/methanol 1:1 (10 mL) were added in a vial. Diazomethane was generated on the interface and injected through a capillary into another vial containing a solution of acid **2a–c** in chloroform (1.5 mg/mL). Methyl esters **3a-c** were prepared in quantitative yields within 15–20 min.

[1] Araki, K.; Welch, J. T. *Tetrahedron Lett.* **1993**, *34*, 2251-2254.
[2] Glastrup, J. *J. Chromatogr. A*, **1998**, *827*, 133-136.

CH_3-C_6H_4-SO_3-N(NO)CH_3 + KOH ⟶ CH_3-C_6H_4-SO_3K + H_2O + **CH_2N_2**

Diazald

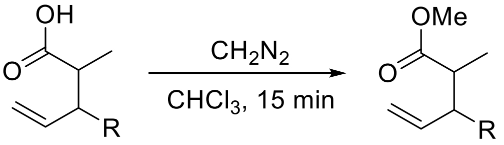

2a-c **3a-c**

Typical procedure for the Ireland–Claisen rearrangement with LDA as a base[3]

n-BuLi in hexanes (*c* 1.6 M, 2.7 mL, 4.32 mmol) was added over 15 min to a solution of dry diisopropylamine (0.47 g, 4.68 mmol) in anhydrous THF (8 mL) at 0 °C. The reaction mixture was stirred for 10 min and then cooled to −78 °C and ester **1a–c** (3.9 mmol) added dropwise within 3 min. The reaction mixture was stirred for another 5 min, then trialkylsilyl chloride (4.3 mmol) was added in one portion. The temperature was allowed to reach ambient temperature and the mixture was stirred at this temperature for 1 h. Then, methanol (1.2 mL) was added and the mixture stirred for another 10 min followed by extraction with 5% NaOH (3 × 10 mL). The aqueous solution was washed with diethyl ether (3 × 12 mL) and acidified with concentrated HCl. The products were isolated by extraction with CH_2Cl_2 (3 × 12 mL) and subsequent solvent evaporation.

Characterization data

2-Methylpent-4-enoic acid (3a)

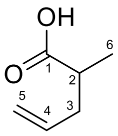

Colourless oil, R_F = 0.38 (hexanes/ethyl acetate 3:1)

^{1}H NMR (600 MHz, $CDCl_3$) δ 1.19 (d, *J* = 7.0 Hz, 3H, **H^6**); 2.21 (dt, *J*= 14.2; 7.2 Hz, 1H, **H^3**); 2.45 (dt, *J*= 13.7; 6.8 Hz, 1H, **H^3**); 2.56 (h, *J*= 7.0 Hz, 1H, **H^2**); 5.06–5.111 (m, 2H, **H^5**); 5.77 (ddt, *J* = 17.1; 10.2; 7.0 Hz, 1H, **H^4**); 11.46 (brs, 1H, **H^{OH}**). ^{1}H NMR spectrum corresponds with those in literature.[4]

2,3-Dimethylpent-4-enoic acid (3b)

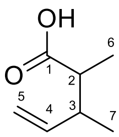

Colourless oil, R_F = 0.05 (hexanes/ethyl acetate 9:1)

^{1}H NMR (600 MHz, $CDCl_3$) δ 1.04 (d, *J* = 6.8 Hz, 0.7×3H, **H^7**); 1.07 (d, *J* = 6.8 Hz, 0.3×3H, **H^7**); 1.13 (d, *J* = 7.0 Hz, 3H, **H^6**); 2.32 –2.37 (m, 0.3×2H, **$H^{2,3}$**);

---

[3] Ireland, R. E.; Mueller, R. H.; Willard, A. K. *J. Am. Chem. Soc.* **1976**, 2868-2877.

[4] Kang, Y-B.; Chen, X-M.; Yao, C-Z.; Ning, X-S. *Chem. Commun.* **2016**, *52*, 6193-6196.

2.44–2.56 (m, 0.7×2H, **H^{2,3}**); 5.01–5.07 (m, 2H, **H^{5}**); 5.66 (ddd, *J* = 17.3; 10.2; 8.3 Hz, 0.3 ×1H, **H^{4}**); 5.78 (ddd, *J* = 17.4; 10.3; 7.4 Hz, 0.7 ×1H, **H^{4}**); 11.59 (brs, 1H, **H^{OH}**). ^{1}H NMR spectrum corresponds with those in literature.[5,6]

^{13}C NMR (151 MHz, $CDCl_3$)

Syn: δ 13.3 (**C^{6}**); 16.2 (**C^{7}**); 40.3 (**C^{3}**); 44.7 (**C^{2}**); 114.8 (**C^{5}**); 140.7 (**C^{4}**); 182.4 (**C^{1}**). ^{13}C NMR spectrum corresponds with those in literature.[5]

Anti: δ 14.5 (**C^{6}**); 18.6 (**C^{7}**); 41.0 (**C^{3}**); 45.1 (**C^{2}**); 115.5 (**C^{5}**); 141.4 (**C^{4}**); 182.7 (**C^{1}**).^{13}C NMR spectrum corresponds with those in literature.[7]

2-Methyl-3-phenylpent-4-enoic acid (3c)

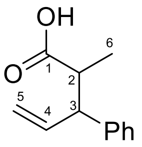

White solid, R_F = 0.05 (hexanes/ethyl acetate 9:1)

^{1}H NMR (600 MHz, $CDCl_3$) δ 1.02 (d, *J* = 6.9 Hz, 0.75×3H, **H^{6}**); 1.26 (d, *J* = 7.0 Hz, 0.25×3H, **H^{6}**); 2.82–2.90 (m, 1H, **H^{2}**); 3.45–3.48 (m, 0.75×1H, **H^{3}**); 3.56 (t, *J* = 9.3 Hz, 0.25×1H, **H^{3}**); 5.05–5.17 (m, 2H, **H^{5}**); 5.94 (m,0.25×1H, **H^{4}**); 6,04 (ddd, *J* = 17.1; 10.2; 8.2 Hz, 0.75×1H, **H^{4}**); 7.19–7.34 (m, 5H, **H^{Ar}**); 1.35 (bs, 1H, **H^{OH}**). ^{1}H NMR spectrum corresponds with those in literature.[8]

^{13}C NMR (151 MHz, $CDCl_3$) δ 15.5 (**C^{6}**); 16.1 (**C^{6}**); 44.9 (**C^{2}**); 45.3 (**C^{2}**); 53.3 (**C^{3}**); 53.4 (**C^{3}**); 115.9 (**C^{5}**); 117.2 (**C^{5}**); 126.8 (**C^{Ar}**); 127.0 (**C^{Ar}**); 127.8 (**C^{Ar}**); 128.3 (**C^{Ar}**); 128.7 (**C^{Ar}**); 128.9 (**C^{Ar}**); 138.3 (**C^{4}**); 139.5 (**C^{4}**); 141.1 (**C^{7}**); 142.2 (**C^{7}**); 182.2 (**C^{1}**); 182.3 (**C^{1}**). ^{13}C NMR spectrum corresponds with those in literature.[8]

2,2-Dimethyl-3-phenylpent-4-enoic acid (3d)

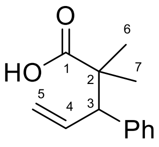

white solid, R_F = 0.35 (hexanes/ethyl acetate 3:1)

^{1}H NMR (600 MHz, $CDCl_3$) δ 1.15 (s, 3H, **H^{6}**); 1.21 (s, 3H, **H^{7}**); 3.65 (d, *J* = 9.5 Hz, 1H, **H^{3}**); 5.15–5.18 (m, 2H, **H^{5}**); 6.26 (dt, *J* = 16.7; 9.9 Hz, 1H, **H^{4}**); 7.22–7.31 (m, 5H, **H^{Ar}**); 11.25–11.84 (bs, 1H, **H^{OH}**). ^{1}H NMR spectrum corresponds with those in literature.[9]

2-Ethyl-3-phenylpent-4-enoic acid (3e)

---

[5] Rye, C. E.; Barker, D. *J. Org. Chem.* **2011**, *76*, 6636-6648.
[6] Ireland, R. E.; Mueller, R. H.; Willard, A. K. *J. Am. Chem. Soc.* **1976**, 2868-2877.
[7] Yodwaree, S.; Soorukram, D.; Kuhakarn, Ch.; Tuchinda, P.; Reutrakul, V.; Pohmakotr, M. *Org. Biomol. Chem.* **2014**, *12*, 6885-6894.
[8] Ishihara, J.; Watanabe, Y.; Koyama, N.; Nishino, Y.; Takahashi, K. *Tetrahedron*, **2011**, *67*, 3659-3667.
[9] Ishihara, J.; Koyama, N.; Nishino, Y.; Takahashi, K.; Hatakeyama, S. *SynLett* **2009**, 2351-2355.

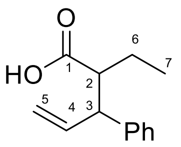

Colorless oil, $R_F = 0.26$ (hexanes/ethyl acetate 3:1)

^{1}H NMR (600 MHz, $CDCl_3$) δ 0.84 (t, *J* = 7.4 Hz, 0.67×3H, **H^7**); 0.96 (t, *J* = 6.2 Hz, 0.33×3H, **H^7**); 1.26–1.33 (m, 0.67×1H, **H^6**); 1.42–1.49 (m, 0.67×1H, **H^6**); 1.59–1.64 (m, 0.33×1H, **H^6**); 1.76–1.82 (m, 0.33×1H, **H^6**); 2.65–2.71 (m, 1H, **H^2**); 3.43–3.46 (m, 0.67×1H, **H^3**); 3.49 (t, *J* = 9.7 Hz, 0.33×1H, **H^3**); 5.0–5.14 (m, 2H, **H^5**); 5.9 (dt, *J* = 16.9; 9.8 Hz, 0.33×1H, **H^4**); 6,0 (ddd, *J* = 17.4; 9.9; 8.8 Hz, 0.67×1H, **H^4**); 7.17–7.33 (m, 5H, **H^{Ar}**); 10.99–11.65 (bs, 1H, **H^{OH}**). ^{1}H NMR spectrum corresponds with those in literature.[10]

(((Z)-1-(cinnamyloxy)prop-1-en-1-yl)oxy)trimethylsilane (**2c**)

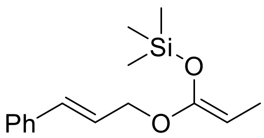

^{1}H NMR (600 MHz, $CDCl_3$) δ 0.09(s, 9H); 1.21 (d, *J*= 7.1 Hz, 3H); 2.10 (q, *J*= 7.1 Hz, 1H); 4.71 (dd, *J*= 6.5; 1.2 Hz, 2H); 6.29 (dt, *J*= 15,9; 6.5 Hz, 1H); 6.65 (d, *J*= 15,9 Hz, 1H); 7.24–7.39 (m, 5H) .

^{13}C NMR (151 MHz, $CDCl_3$) δ 2.6; 11.2; 30.5; 64.7; 124.1; 126.8; 128.2; 128.8; 134.0; 136.6; 176.3.

Pictures of ^{1}H and ^{13}C NMR spectra

^{1}H NMR of **3a**

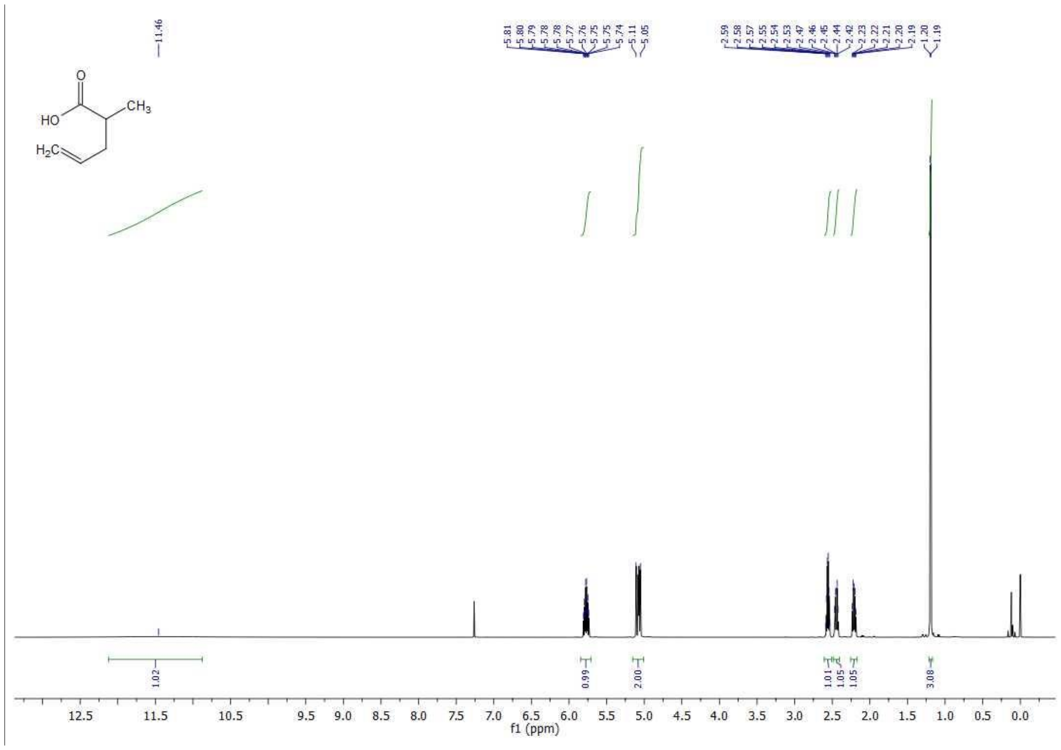

[10] Eriksson, M.; Hjelmencrantz, A.; Nilsson, M.; Olsson, T. *Tetrahedron* **1995**, *51*, 12631-12644.

^{1}H NMR of **3b**

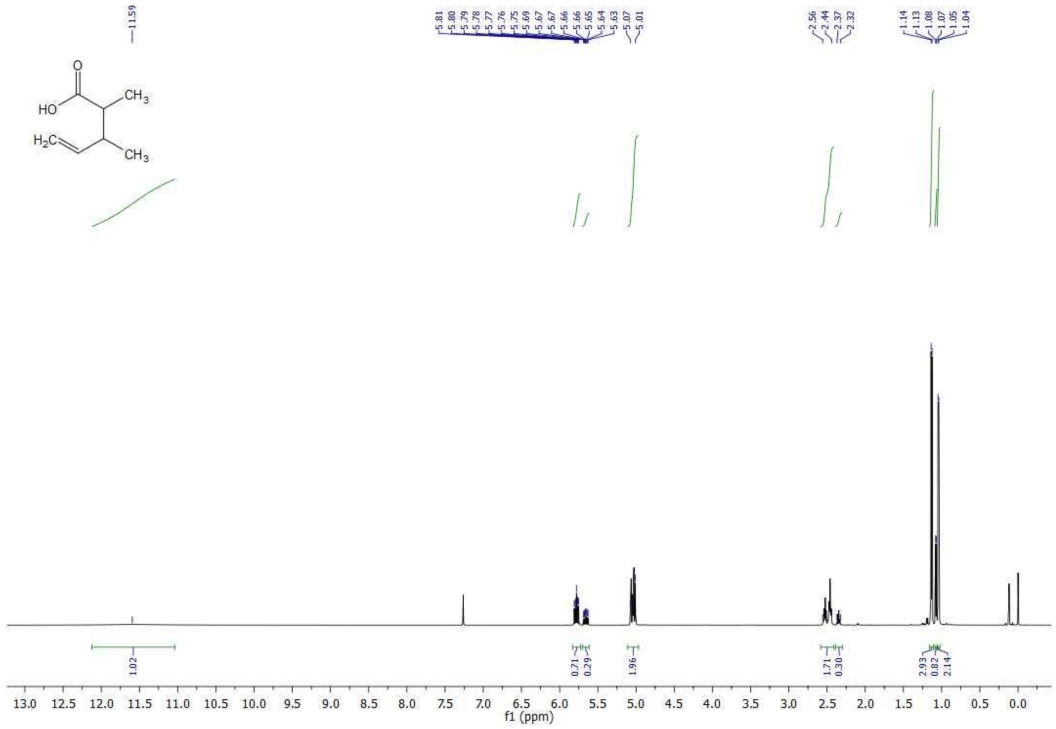

^{13}C NMR of **3b**

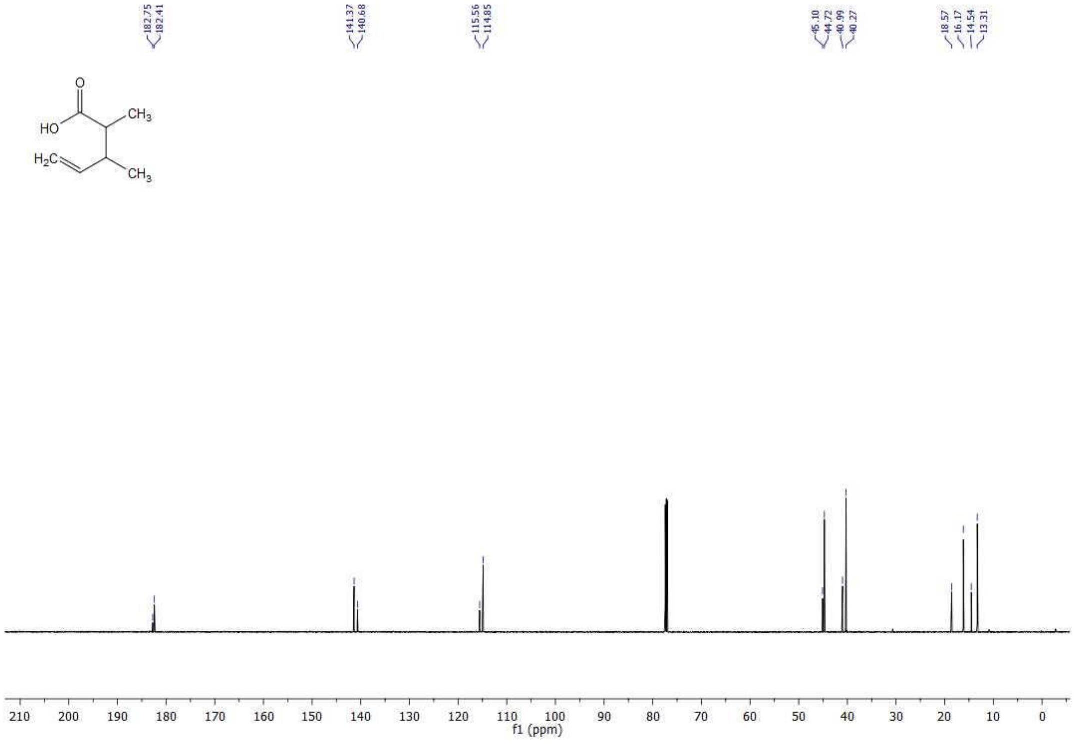

^{1}H NMR of **3c**

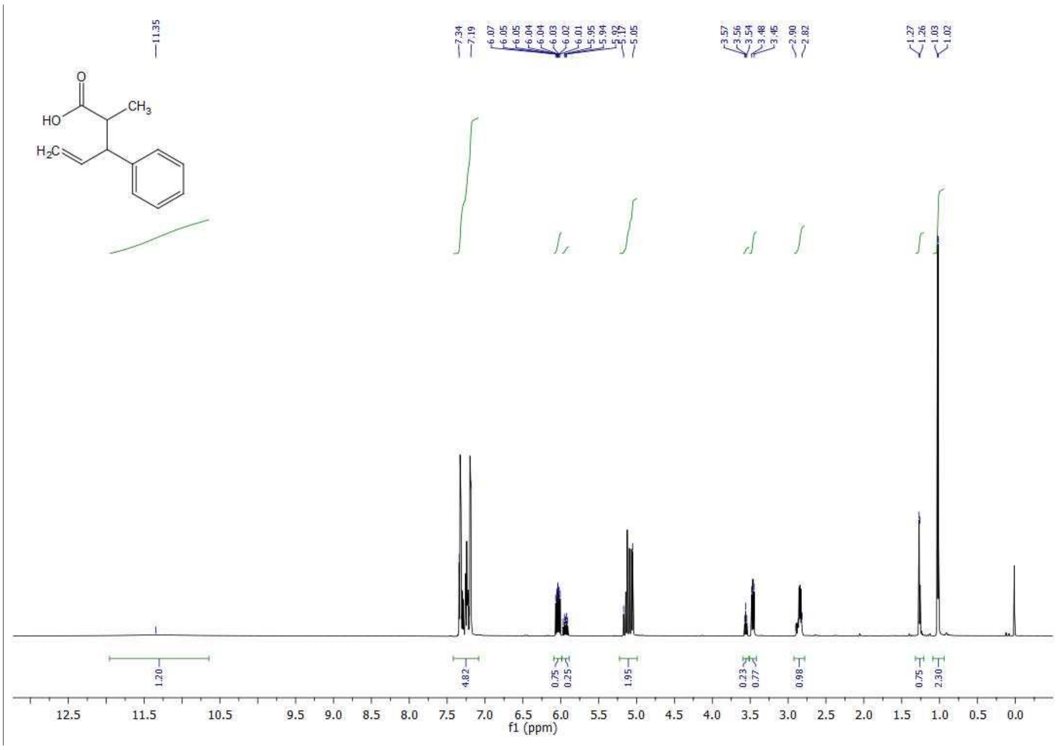

^{13}C NMR of **3c**

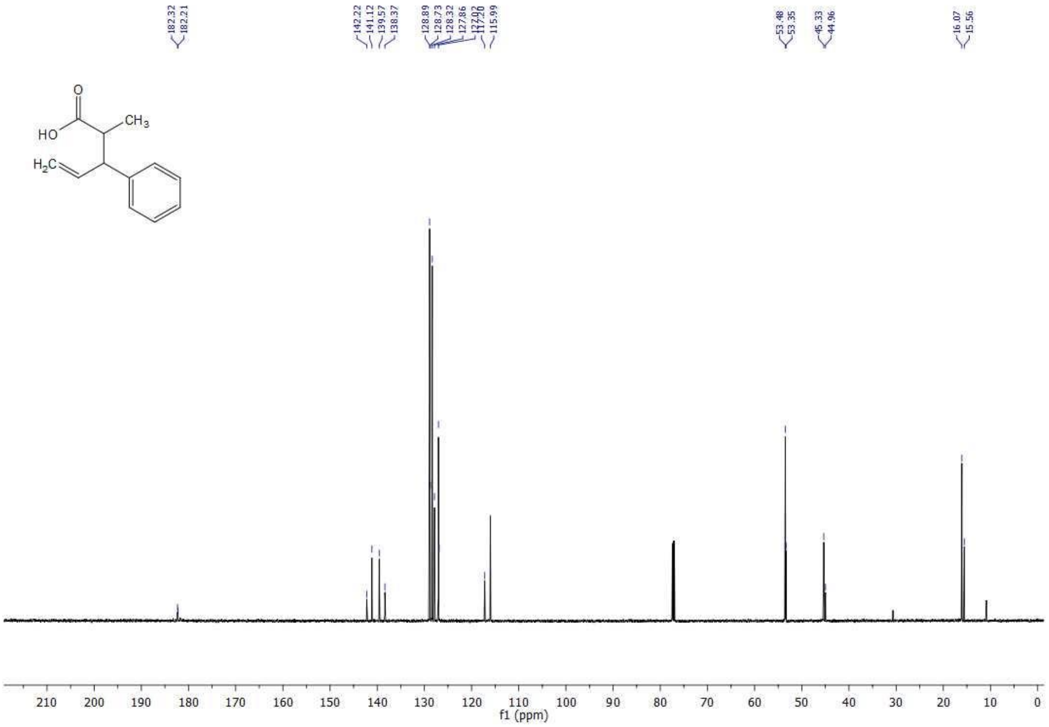

^{1}H NMR of **3d**

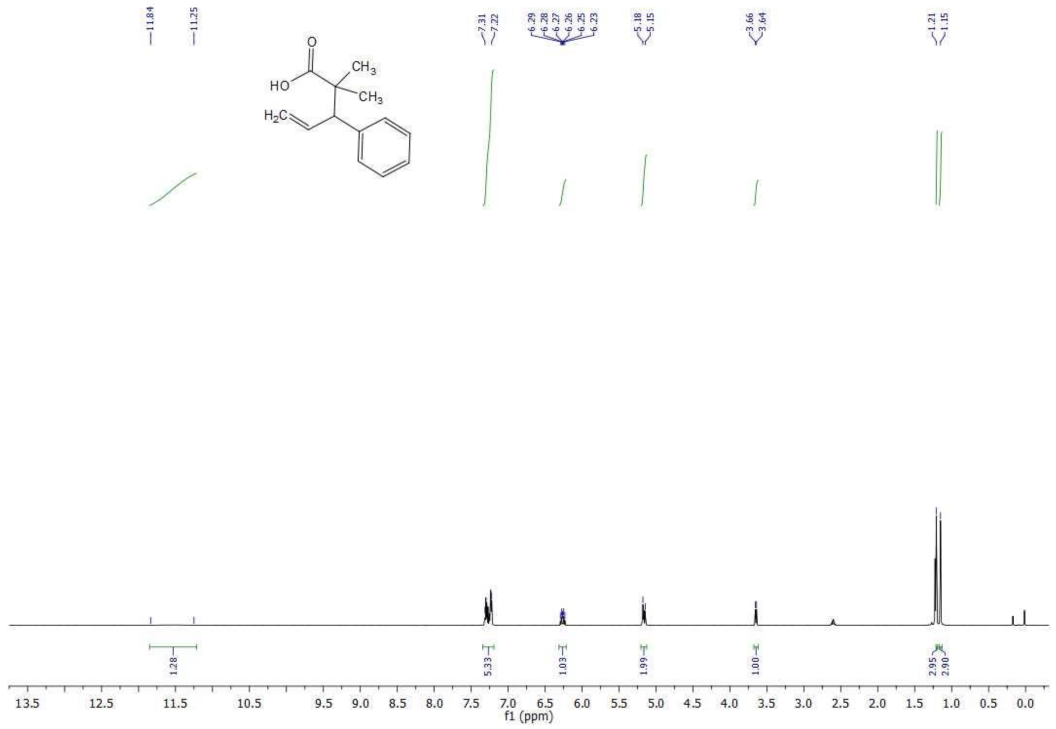

^{1}H NMR of **3e**

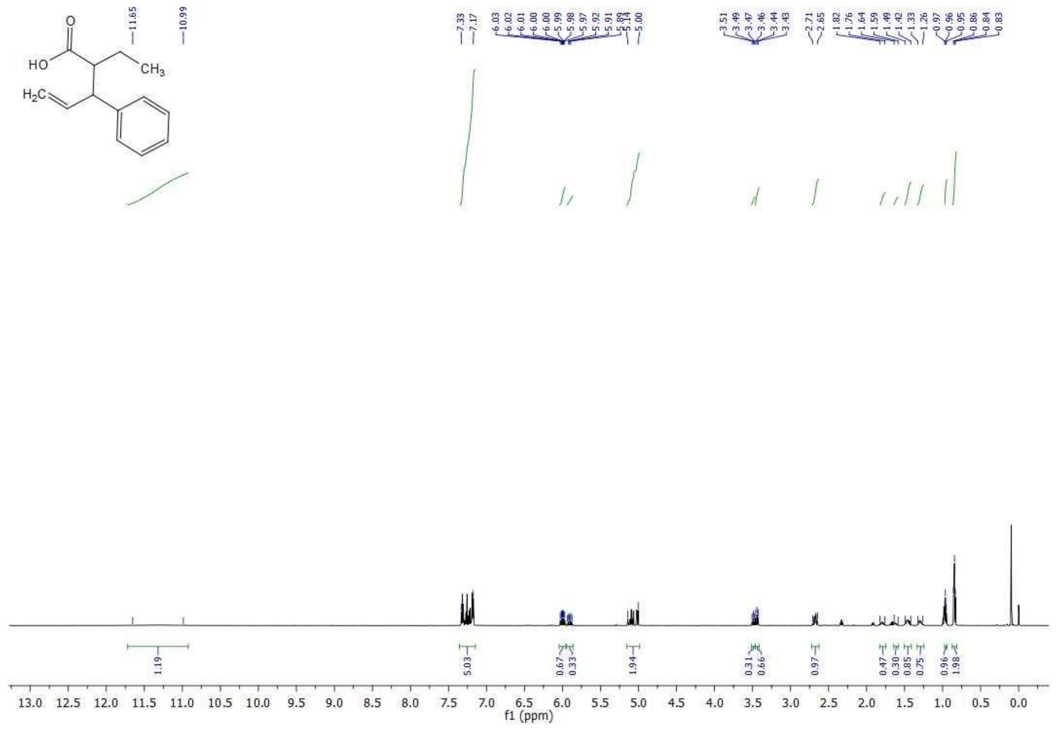

^{1}H NMR of **2c**

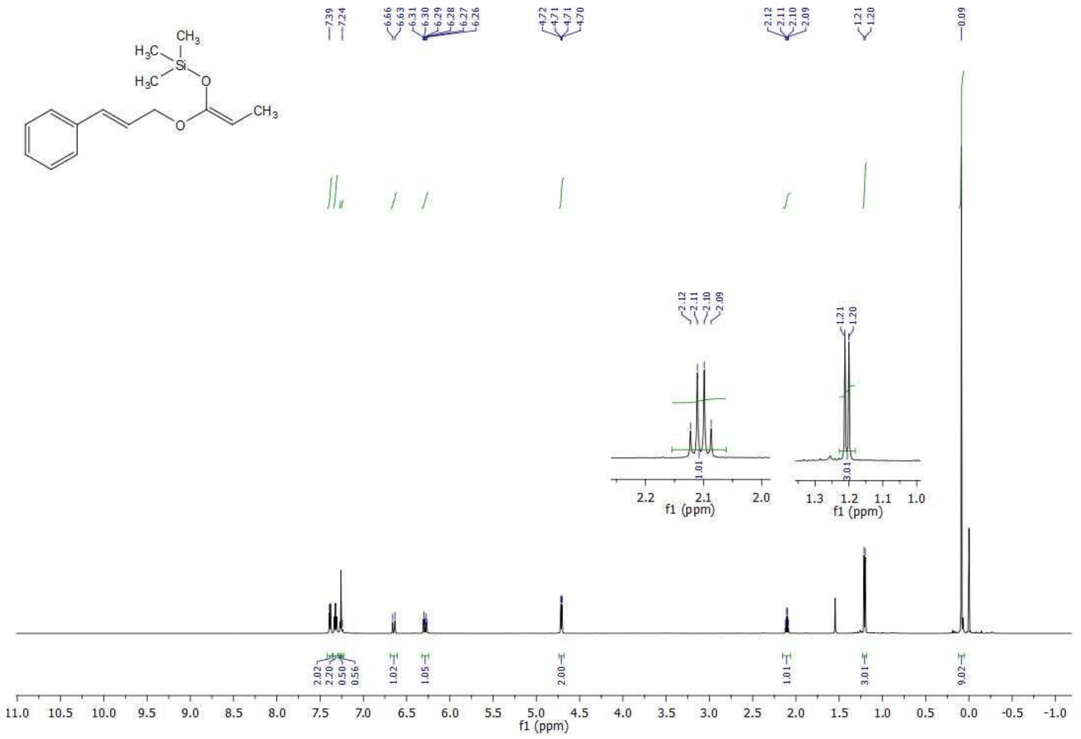

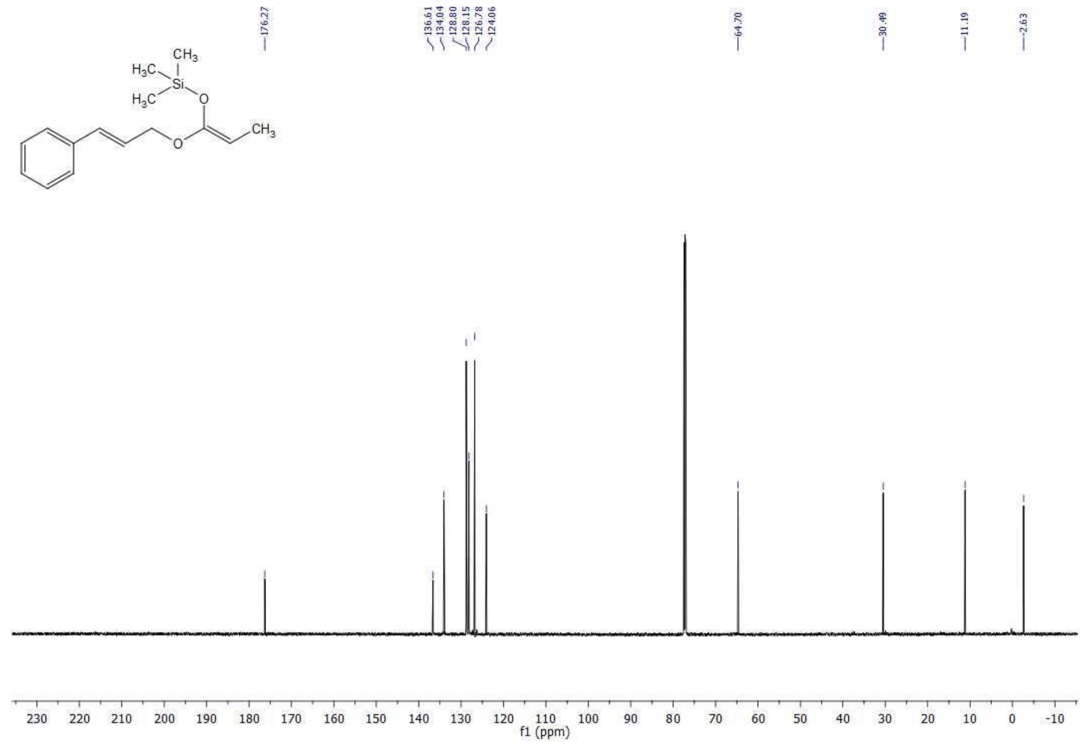

Computational details

The molecular models were built and optimized by AM1 method in Spartan 18 program package.[11] From the conformer distribution, several of the most stable conformers were selected and pre-optimized in Spartan at HF/3-21G level and then fully geometrically optimized at ωB97X-D/6-31G* level.[12] Transition states were optimized by ωB97X-D/6-31G* and were verified by visualization of their imaginary frequencies.

(*E*)-Silyl ketene acetal 2c

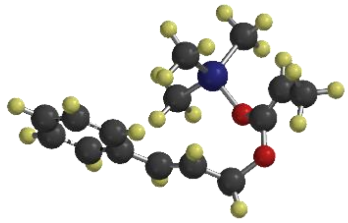

Method: ωB97X-D
Basis set: 6-31G*
Energy: -1024.571296 hartrees
Zero Point Energy : 839.22 kJ/mol (ZPE)
Temperature Correction : 49.83 kJ/mol (vibration + gas law + rotation + translation)
Enthalpy Correction : 889.04 kJ/mol (ZPE + temperature correction)
Enthalpy : -1024.232678 au (Electronic Energy + Enthalpy Correction)
Entropy : 674.59 J/mol•K
Gibbs Energy : -1024.309284 au (Enthalpy - T*Entropy)
Cv : 341.83 J/mol•K

Coordinates:

C	-0.041081	-1.472650	-2.159880	C	-1.928051	0.380800	0.626803
C	-2.254786	-1.054198	-1.385021	H	-3.192666	-1.616749	-1.431614
C	-1.850092	-0.814717	0.038777	H	-1.475962	-1.689001	0.568453

---

[11] Spartan ´18, Wavefunction, Inc., Irvine, CA

[12] Shao, Y.; Gan, Z.; Epifanovsky, E.; Gilbert, A. T. B.; Wormit, M.; Kussmann, J.; Lange, A. W.; Behn, A.; Deng, J.; Feng, X.; Ghosh, D.; Goldey, M.; Horn, P. R.; Jacobson, L. D.; Kaliman, I.; Khaliullin, R. Z.; Kuś, T.; Landau, A.; Liu, J.; Proynov, E. I.; Rhee, Y. M.; Richard, R. M.; Rohrdanz, M. A.; Steele, R. P.; Sundstrom, E. J.; Woodcock, H. L.; Zimmerman, P. M.; Zuev, D.; Albrecht, B.; Alguire, E.; Austin, B.; Beran, G. J. O.; Bernard, Y. A.; Berquist, E.; Brandhorst, K.; Bravaya, K. B.; Brown, S. T.; Casanova, D.; Chang, C.-M.; Chen, Y.; Chien, S. H.; Closser, K. D.; Crittenden, D. L.; Diedenhofen, M.; DiStasio, R. A.; Do, H.; Dutoi, A. D.; Edgar, R. G.; Fatehi, S.; Fusti-Molnar, L.; Ghysels, A.; Golubeva-Zadorozhnaya, A.; Gomes, J.; Hanson-Heine, M. W. D.; Harbach, P. H. P.; Hauser, A. W.; Hohenstein, E. G.; Holden, Z. C.; Jagau, T.-C.; Ji, H.; Kaduk, B.; Khistyaev, K.; Kim, J.; Kim, J.; King, R. A.; Klunzinger, P.; Kosenkov, D.; Kowalczyk, T.; Krauter, C. M.; Lao, K. U.; Laurent, A. D.; Lawler, K. V.; Levchenko, S. V.; Lin, C. Y.; Liu, F.; Livshits, E.; Lochan, R. C.; Luenser, A.; Manohar, P.; Manzer, S. F.; Mao, S.-P.; Mardirossian, N.; Marenich, A. V.; Maurer, S. A.; Mayhall, N. J.; Neuscamman, E.; Oana, C. M.; Olivares-Amaya, R.; O'Neill, D. P.; Parkhill, J. A.; Perrine, T. M.; Peverati, R.; Prociuk, A.; Rehn, D. R.; Rosta, E.; Russ, N. J.; Sharada, S. M.; Sharma, S.; Small, D. W.; Sodt, A. *Mol. Phys.* **2015**, *113*, 184-215.

H	-2.396021	-0.104209	-1.911471	H	3.527222	1.615987	-2.210073
H	-2.292496	1.216951	0.028691	H	1.009575	0.039061	0.912856
O	0.098773	-0.130618	-2.186621	H	2.128295	-1.037121	0.054774
Si	1.320020	0.783161	-1.439204	H	2.677266	0.554771	0.613772
C	2.783081	0.915464	-2.607363	H	-1.942081	2.819595	1.684015
C	0.545499	2.467068	-1.173907	H	-1.256529	3.513746	3.952492
C	1.828667	0.006904	0.187263	H	3.281040	-0.048677	-2.752692
C	-1.537301	0.730870	2.002127	H	-1.029874	-1.263044	2.661723
C	-0.753129	1.518866	4.583444	H	-0.343121	-0.569065	4.914661
C	-1.591014	2.073889	2.393831	H	-0.451507	1.820186	5.581935
C	-1.081259	-0.212842	2.933476	O	-1.334391	-1.884165	-2.085744
C	-0.692875	0.177856	4.208121	C	0.950140	-2.365873	-2.257313
C	-1.204276	2.466492	3.670691	C	0.765034	-3.851660	-2.176783
H	-0.237034	2.412776	-0.410378	H	1.949386	-1.984882	-2.443548
H	0.100975	2.853156	-2.097300	H	-0.265150	-4.104345	-1.914663
H	2.468781	1.280302	-3.590993	H	0.995289	-4.342164	-3.131182
H	1.293886	3.190817	-0.830801	H	1.427754	-4.292823	-1.421413

(*Z*)-Silyl ketene acetal 2c

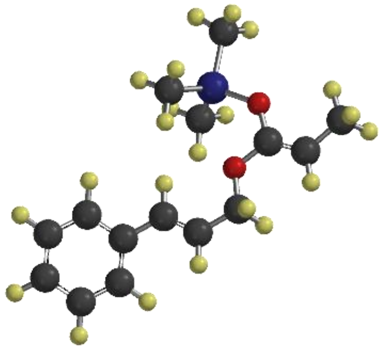

Method: ωB97X-D
Basis set: 6-31G*
Energy: -1024.571293 hartrees
Zero Point Energy : 838.41 kJ/mol (ZPE)
Temperature Correction : 49.89 kJ/mol (vibration + gas law + rotation + translation)
Enthalpy Correction : 888.30 kJ/mol (ZPE + temperature correction)
Enthalpy : -1024.232958 au (Electronic Energy + Enthalpy Correction)
Entropy : 660.13 J/mol•K
Gibbs Energy : -1024.307922 au (Enthalpy - T*Entropy)
Cv : 342.24 J/mol•K

Coordinates:

C	-0.950121	-1.792969	-1.668624	H	-2.661619	-1.341016	0.321812
C	-2.184522	-0.458813	-0.131736	H	-2.875732	0.920138	1.408801
C	-1.966038	0.598556	0.904696	H	-2.866888	-0.089303	-0.912808
C	-0.782146	1.120201	1.228855	H	0.101905	0.742063	0.721796

O	0.299660	-2.025019	-2.096409	H	1.581544	0.732020	-3.030366
Si	1.730447	-1.271617	-1.576063	H	0.840863	1.059927	-1.457053
C	2.034338	-1.620244	0.242549	H	2.607467	1.052114	-1.624136
C	3.036059	-2.111942	-2.622023	H	1.557845	1.678388	2.366086
C	1.681304	0.565244	-1.952123	H	2.059123	3.374018	4.091782
C	-0.535294	2.156937	2.245561	H	1.206254	-1.272621	0.866747
C	0.032193	4.089765	4.204669	H	-2.550572	2.921269	2.340380
C	0.763081	2.317488	2.743705	H	-2.056308	4.595755	4.072518
C	-1.541785	2.998491	2.735960	H	0.249491	4.837420	4.961527
C	-1.262079	3.951066	3.707565	O	-0.953665	-0.826820	-0.708820
C	1.045359	3.270581	3.716142	C	-2.017307	-2.443035	-2.146239
H	2.853036	-1.944903	-3.688653	C	-1.929906	-3.500135	-3.207439
H	3.044812	-3.192911	-2.447460	H	-2.318800	-4.460878	-2.846960
H	2.152982	-2.696138	0.412019	H	-0.895691	-3.653127	-3.524267
H	4.034253	-1.724753	-2.387119	H	-2.517993	-3.226906	-4.092710
H	2.952802	-1.126504	0.582285	H	-2.998335	-2.201784	-1.754450

TS-(*E*)-uncatalyzed

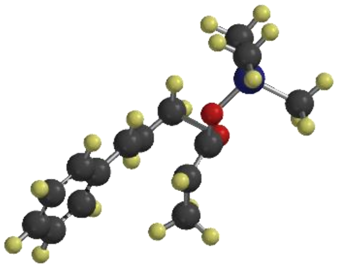

Method: ωB97X-D
Basis set: 6-31G*
Energy: -1024.537184 hartrees

Imaginary vibration: -504 cm^{-1} (Uncorrected); -475 cm^{-1} (Corrected); Intensity 25.81

Zero Point Energy : 835.69 kJ/mol (ZPE)
Temperature Correction : 50.40 kJ/mol (vibration + gas law + rotation + translation)
Enthalpy Correction : 886.09 kJ/mol (ZPE + temperature correction)
Enthalpy : -1024.199690 au (Electronic Energy + Enthalpy Correction)
Entropy : 634.87 J/mol•K
Gibbs Energy : -1024.271785 au (Enthalpy - T*Entropy)
Cv : 345.44 J/mol•K

Coordinates:

C	-0.654372	0.970672	0.263163	H	0.269753	0.184463	3.357203
C	0.514761	-0.025028	2.321985	H	-0.435852	-1.945538	2.335506
C	0.211184	-1.276493	1.775401	H	1.371936	0.519695	1.938772
C	0.423663	-1.480592	0.423500	H	1.144238	-0.829471	-0.068194

O	0.320705	1.627286	-0.432874	H	-1.302296	4.312535	-0.642586
Si	0.840443	3.195551	-0.048692	H	-1.239603	3.927389	1.086617
C	1.970911	3.209186	1.448855	H	-0.343929	5.321635	0.454111
C	1.791578	3.718027	-1.576282	H	1.301795	-2.111653	-2.008104
C	-0.651912	4.291961	0.238572	H	0.816737	-4.138038	-3.334438
C	0.096473	-2.703016	-0.327951	H	1.421124	2.936476	2.354831
C	-0.467448	-5.002898	-1.837478	H	-1.202475	-3.584629	1.153959
C	0.648931	-2.880743	-1.602951	H	-1.689283	-5.598177	-0.168632
C	-0.751273	-3.698961	0.173448	H	-0.685237	-5.893057	-2.419612
C	-1.029371	-4.837293	-0.574345	O	-0.734091	1.160081	1.526420
C	0.373789	-4.018700	-2.350229	C	-1.316272	-0.044958	-0.395147
H	1.145461	3.713699	-2.460429	C	-2.519523	-0.719680	0.183044
H	2.629783	3.040361	-1.770928	H	-1.154197	-0.123379	-1.466592
H	2.809455	2.513826	1.329110	H	-2.575659	-1.766784	-0.135774
H	2.198320	4.729124	-1.459677	H	-2.499554	-0.676527	1.275033
H	2.392359	4.211007	1.595324	H	-3.441049	-0.227361	-0.153937

TS-(*Z*)-uncatalyzed

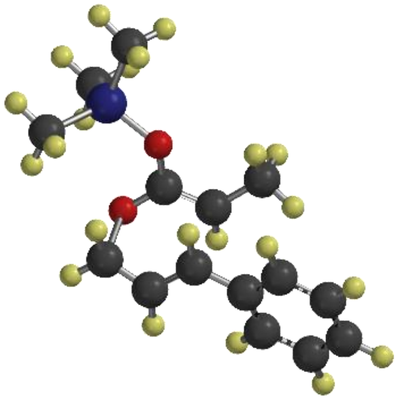

Method: ωB97X-D
Basis set: 6-31G*
Energy: -1024.538466 hartrees

Imaginary vibration: -532 cm^{-1} (Uncorrected); -501 cm^{-1} (Corrected); Intensity 36.89

Zero Point Energy : 835.02 kJ/mol (ZPE)
Temperature Correction : 50.43 kJ/mol (vibration + gas law + rotation + translation)
Enthalpy Correction : 885.45 kJ/mol (ZPE + temperature correction)
Enthalpy : -1024.201216 au (Electronic Energy + Enthalpy Correction)
Entropy : 642.16 J/mol•K
Gibbs Energy : -1024.274139 au (Enthalpy - T*Entropy)
Cv : 345.87 J/mol•K

Coordinates:

C	-0.701572	0.917252	0.370373	C	0.437292	-0.070351	2.450043

C	0.117906	-1.331638	1.937587
C	0.336295	-1.577471	0.593505
H	0.202660	0.167360	3.481422
H	-0.552872	-1.972438	2.503252
H	1.301559	0.452618	2.051361
H	1.105622	-0.988099	0.096543
O	0.225507	1.667654	-0.291016
Si	0.676840	3.232813	0.183705
C	1.818466	3.204275	1.672406
C	1.596049	3.880875	-1.315559
C	-0.857356	4.248738	0.536165
C	-0.036719	-2.815651	-0.112814
C	-0.694105	-5.153013	-1.520178
C	0.695322	-3.204827	-1.239912
C	-1.117935	-3.611069	0.287415
C	-1.440773	-4.769182	-0.407554
C	0.374496	-4.365375	-1.935782
H	0.947651	3.892851	-2.197917
H	2.464810	3.255498	-1.547331
H	2.671751	2.535695	1.511674
H	1.954534	4.902506	-1.145980
H	2.217685	4.207776	1.862380
H	-1.512790	4.289095	-0.340489
H	-1.424652	3.815963	1.365539
H	-0.589728	5.277419	0.803965
H	1.526980	-2.588522	-1.570579
H	0.958495	-4.651968	-2.804952
H	1.285105	2.875455	2.569316
H	-1.721814	-3.311143	1.138683
H	-2.283862	-5.372426	-0.085005
H	-0.950312	-6.057264	-2.063268
O	-0.816159	1.063715	1.638166
C	-1.286541	-0.128381	-0.315232
C	-1.223754	-0.285553	-1.803402
H	-1.315803	-1.339695	-2.084218
H	-0.280815	0.101680	-2.198874
H	-2.040218	0.258499	-2.295506
H	-2.067248	-0.653672	0.222063

(*E*)-Silyl ketene acetal + Schreiner thiourea

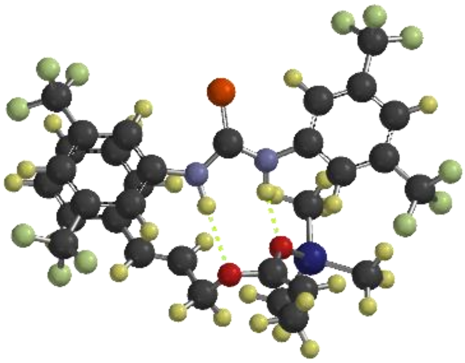

Method: ωB97X-D
Basis set: 6-31G*
Energy: -3382.515231 hartrees

Zero Point Energy : 1456.94 kJ/mol (ZPE)
Temperature Correction : 110.38 kJ/mol (vibration + gas law + rotation + translation)
Enthalpy Correction : 1567.31 kJ/mol (ZPE + temperature correction)
Enthalpy : -3381.918273 au (Electronic Energy + Enthalpy Correction)
Entropy : 1302.40 J/mol•K
Gibbs Energy : -3382.066173 au (Enthalpy - T*Entropy)
Cv : 807.25 J/mol•K

Energy (ωB97X-D/6-31G*//M06-2X/6-311+G**): -3383.12863 au

[E(E-enol): -1024.67643 au; E(**C12**): -2358.40134 au]

Coordinates:

C	0.048666	2.554667	1.133876
C	-0.500561	0.989848	2.834876
C	-0.062715	-0.391684	2.472813
C	-0.759169	-1.479176	2.815643
H	-1.385187	0.960931	3.476395
H	0.832374	-0.466214	1.860390
H	0.282084	1.555568	3.352092
H	-1.653060	-1.348481	3.425244
O	1.128530	1.858712	0.679676
Si	2.794316	2.072596	0.999544
C	3.564476	0.785717	-0.113556
C	3.356631	3.804720	0.581107
C	3.080078	1.717513	2.816565
C	-0.494005	-2.865203	2.404757
C	-0.166467	-5.523381	1.576848
C	-1.269149	-3.888695	2.958996
C	0.450406	-3.197136	1.423854
C	0.611313	-4.512630	1.014201
C	-1.105651	-5.208354	2.552186
H	2.896962	4.549616	1.238712
H	3.138792	4.077482	-0.454821
H	3.359710	1.002511	-1.167665
H	4.443115	3.874322	0.716115
H	4.651883	0.755755	0.016119
H	2.562050	2.447457	3.448996
H	2.737408	0.715843	3.097419
H	4.148360	1.783092	3.052585
H	-2.026509	-3.640525	3.697772
H	-1.727546	-5.987121	2.981721
H	3.174768	-0.214341	0.106313
H	1.038432	-2.417518	0.947757
H	1.333147	-4.750115	0.238574
H	-0.052274	-6.548651	1.239650
O	-0.904748	1.739048	1.661719
C	-0.137534	3.864957	0.971118
C	-0.799157	-0.398008	-1.788576
S	-1.286254	-1.145732	-3.195163
N	-1.364237	-0.606339	-0.555274
N	0.197914	0.531924	-1.696972
C	-2.281702	-1.589950	-0.155905
C	-3.986975	-3.519191	0.941126
C	-2.230773	-2.908922	-0.612488
C	-3.189624	-1.242816	0.840691
C	-4.020781	-2.210201	1.395621
C	-3.091505	-3.851451	-0.072303
C	0.854360	1.235713	-2.723494
C	2.277663	2.760686	-4.586760
C	1.061160	2.600342	-2.509269
C	1.363347	0.630835	-3.871641
C	2.060570	1.404006	-4.794920
C	1.776213	3.350168	-3.431741
C	2.558199	0.769881	-6.068509
C	-3.076671	-5.261881	-0.598260
C	-4.877496	-1.838991	2.571257
C	2.054820	4.803724	-3.166804
F	3.713536	1.326433	-6.473913
F	1.898733	5.553298	-4.265624
F	3.325051	4.984352	-2.747720
F	2.772859	-0.545689	-5.921700
F	1.255267	5.307877	-2.210666
F	1.674379	0.923801	-7.066481
F	-1.908800	-5.576178	-1.178089
F	-4.039599	-5.449187	-1.516358
F	-3.295203	-6.157740	0.382609
F	-4.156113	-1.854660	3.719145
F	-5.900343	-2.684344	2.747663
F	-5.380664	-0.600577	2.457477
H	-1.511032	-3.195702	-1.365721
H	-3.226296	-0.219240	1.200715
H	-4.633775	-4.272812	1.374175
H	0.434756	0.879851	-0.768735
H	-1.212906	0.116287	0.143914
H	1.212474	-0.427058	-4.037991
H	2.830753	3.347649	-5.311136
H	0.644500	3.069778	-1.624426
H	0.625716	4.411718	0.425356
C	-1.340075	4.618907	1.450633
H	-2.010428	3.972850	2.021752
H	-1.045741	5.462678	2.085229
H	-1.903257	5.032462	0.606183

(*Z*)-Silyl ketene acetal + Schreiner thiourea

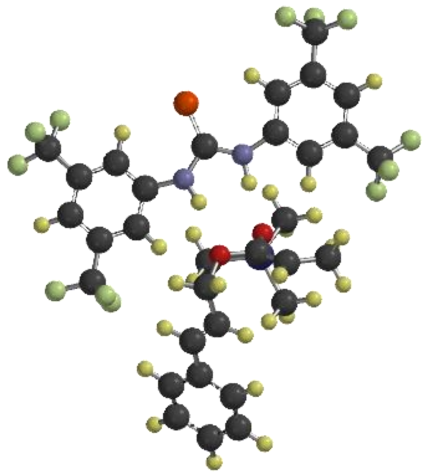

Method: ωB97X-D
Basis set: 6-31G*
Energy: -3382.508748 hartrees

Zero Point Energy : 1455.81 kJ/mol (ZPE)
Temperature Correction : 110.41 kJ/mol (vibration + gas law + rotation + translation)
Enthalpy Correction : 1566.22 kJ/mol (ZPE + temperature correction)
Enthalpy : -3381.912205 au (Electronic Energy + Enthalpy Correction)
Entropy : 1292.42 J/mol•K
Gibbs Energy : -3382.058971 au (Enthalpy - T*Entropy)
Cv : 807.58 J/mol•K

Energy (ωB97X-D/6-31G*//M06-2X/6-311+G**): -3383.12077 au
[E(Z-enol): -1024.67962 au; E(**C12**): -2358.39846 au]

Coordinates:

C	0.558330	-1.649499	-0.670085
C	2.867980	-1.229669	-0.121704
C	2.813914	-1.968589	1.181846
C	3.007235	-1.335894	2.343241
H	3.469825	-0.322962	-0.034941
H	2.524520	-3.015485	1.148957
H	3.280142	-1.835684	-0.935461
H	3.284227	-0.283850	2.307636
O	-0.628008	-1.060699	-0.379116
Si	-1.259873	-0.883687	1.215785
C	-0.440724	0.573166	2.052732
C	-3.066651	-0.575450	0.854803
C	-0.926064	-2.477983	2.133061
C	2.852196	-1.878875	3.700841
C	2.520058	-2.784080	6.332934
C	2.981514	-0.999824	4.782470
C	2.553326	-3.222766	3.960813
C	2.389298	-3.670372	5.264775
C	2.816322	-1.447093	6.088340
H	-3.193315	0.327864	0.246580
H	-3.647023	-0.440092	1.773833
H	-0.565704	1.494145	1.473183
H	-3.503184	-1.413939	0.301190
H	-0.884007	0.740772	3.041294
H	-1.303961	-3.346054	1.583350
H	0.148245	-2.612383	2.296154
H	-1.409344	-2.463360	3.116755
H	3.209937	0.044765	4.589287
H	2.920940	-0.750499	6.914296
H	0.629363	0.397611	2.199293
H	2.454633	-3.927213	3.139908
H	2.160857	-4.715557	5.451224

H	2.390737	-3.137530	7.351149
O	1.563635	-0.742586	-0.492355
C	0.671620	-2.894908	-1.130094
C	-0.479357	-3.835822	-1.330472
H	-0.298724	-4.789813	-0.822753
H	-0.629739	-4.056103	-2.393544
H	-1.417066	-3.425578	-0.948111
H	1.660800	-3.251073	-1.401380
C	-0.826441	2.139104	-2.194320
S	-1.338894	3.531339	-2.950177
N	0.250393	2.044103	-1.341091
N	-1.410219	0.907752	-2.315420
C	1.041333	3.005086	-0.696516
C	2.735067	4.710982	0.771213
C	1.006469	4.385752	-0.910289
C	1.932982	2.493894	0.253854
C	2.764132	3.337106	0.973787
C	1.849697	5.212893	-0.174022
C	-2.453489	0.405104	-3.106917
C	-4.476813	-0.895558	-4.559605
C	-2.823922	-0.912030	-2.805566
C	-3.106518	1.071804	-4.145142
C	-4.102869	0.408056	-4.858636
C	-3.825669	-1.546870	-3.517829
C	-4.830426	1.146469	-5.955047
C	1.841252	6.691550	-0.472562
C	3.673217	2.721526	1.998236
C	-4.142524	-2.979523	-3.187282
F	-5.301272	0.301573	-6.888649
F	-5.381074	-3.321554	-3.565700
F	-4.043204	-3.216645	-1.862681
F	-5.882872	1.828351	-5.472429
F	-3.291062	-3.827600	-3.791914
F	-4.035748	2.032237	-6.571985
F	0.605345	7.140322	-0.732548
F	2.600892	6.969905	-1.545074
F	2.326255	7.409909	0.554451
F	2.969075	2.154831	3.003080
F	4.512183	3.603442	2.547116
F	4.420053	1.731860	1.461244
H	0.326950	4.804916	-1.637909
H	1.951952	1.424900	0.439398
H	3.381264	5.372569	1.334121
H	-1.088422	0.210290	-1.645411
H	0.535925	1.099722	-1.096658
H	-2.838652	2.089087	-4.390803
H	-5.254576	-1.392641	-5.125380
H	-2.316418	-1.436329	-2.002856

TS-(*E*)-Schreiner thiourea-catalyzed

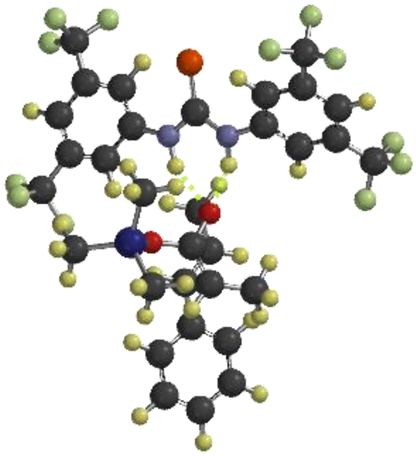

Method: ωB97X-D

Basis set: 6-31G*

Energy: -3382.470065 hartrees

Imaginary vibration: -408 cm^{-1} (Uncorrected); -385 cm^{-1} (Corrected); Intensity 37.41

Zero Point Energy : 1448.89 kJ/mol (ZPE)

Temperature Correction : 111.19 kJ/mol (vibration + gas law + rotation + translation)
Enthalpy Correction : 1560.08 kJ/mol (ZPE + temperature correction)
Enthalpy : -3381.875861 au (Electronic Energy + Enthalpy Correction)
Entropy : 1300.59 J/mol•K
Gibbs Energy : -3382.023555 au (Enthalpy - T*Entropy)
Cv : 813.30 J/mol•K

Energy (ωB97X-D/6-31G*//M06-2X/6-311+G**): -3383.08386 au
[E(TS-E-enol): -1024.64305 au; E(**C12**): -2358.40080 au]

Coordinates:

C	-0.326768	-0.991586	-1.033402
C	-0.060922	-1.803823	1.482695
C	-0.498567	-3.031849	0.991603
C	0.096567	-3.537544	-0.147685
H	-0.557516	-1.336695	2.325649
H	-1.437120	-3.439686	1.354434
H	0.976553	-1.519383	1.338501
H	1.051213	-3.102044	-0.439151
O	0.956300	-0.718604	-1.422850
Si	1.396139	0.351489	-2.674022
C	1.100958	2.100071	-2.072734
C	3.202927	-0.013012	-2.970483
C	0.373202	-0.019018	-4.201608
C	-0.255191	-4.772573	-0.853079
C	-0.834248	-7.121034	-2.269082
C	0.574790	-5.204912	-1.896856
C	-1.384178	-5.538361	-0.532548
C	-1.668806	-6.702828	-1.235574
C	0.290585	-6.368181	-2.598784
H	3.339878	-1.045250	-3.310447
H	3.807133	0.122567	-2.069131
H	1.755098	2.349555	-1.230093
H	3.607512	0.649814	-3.743821
H	1.295404	2.824847	-2.872065
H	0.531120	-1.046187	-4.548887
H	-0.698195	0.116143	-4.020708
H	0.660369	0.653990	-5.018033
H	1.451477	-4.615860	-2.154692
H	0.946875	-6.689690	-3.401420
H	0.065034	2.242996	-1.742800
H	-2.042504	-5.230473	0.273707
H	-2.544867	-7.287976	-0.973933
H	-1.058884	-8.032136	-2.814800
O	-0.685466	-0.518617	0.111058
C	-1.051491	-1.936324	-1.713598
C	-2.482907	-2.243260	-1.414616
H	-0.634749	-2.312028	-2.643348
H	-2.666321	-3.323827	-1.453378
H	-2.765390	-1.868701	-0.428168
H	-3.145192	-1.779742	-2.156299
C	0.071655	2.537481	1.549424
S	0.424161	3.942418	2.374050
N	-1.125620	2.256748	0.937368
N	0.929417	1.486343	1.368401
C	-2.327598	2.982620	0.942946
C	-4.820104	4.261920	0.862604
C	-2.397079	4.375812	0.900752
C	-3.508215	2.236514	0.919989
C	-4.740143	2.874549	0.875441
C	-3.640807	4.996650	0.873969
C	2.236691	1.262208	1.816906
C	4.824633	0.528454	2.623336
C	2.974967	0.326405	1.084795
C	2.799270	1.826946	2.964218
C	4.084896	1.458288	3.344966
C	4.249385	-0.039018	1.493391
H	-1.490815	4.964208	0.889737
H	-3.459024	1.151748	0.934549
H	-5.781309	4.759782	0.828052
H	0.572882	0.744006	0.764600
H	-1.223159	1.301311	0.596638
H	2.235137	2.535155	3.554049
H	5.817360	0.239920	2.945070
H	2.534888	-0.130040	0.201922
C	4.692365	2.111232	4.560713
C	5.012783	-1.030333	0.660064
F	3.769942	2.360840	5.502217
F	5.639990	1.337590	5.117235

F	5.573835	-0.441363	-0.414909	F	-3.661311	6.970746	2.156500
F	4.207909	-2.003333	0.190953	F	-4.831131	6.963938	0.338857
F	6.001535	-1.617727	1.347592	F	-7.023242	2.675545	0.313408
F	5.269708	3.282466	4.248269	F	-5.831186	0.864981	0.292990
C	-3.700013	6.502381	0.898897	F	-6.376042	1.782058	2.171742
C	-5.997735	2.048813	0.909957	F	-2.668732	7.051471	0.238786

TS-(*Z*)-Schreiner thiourea-catalyzed

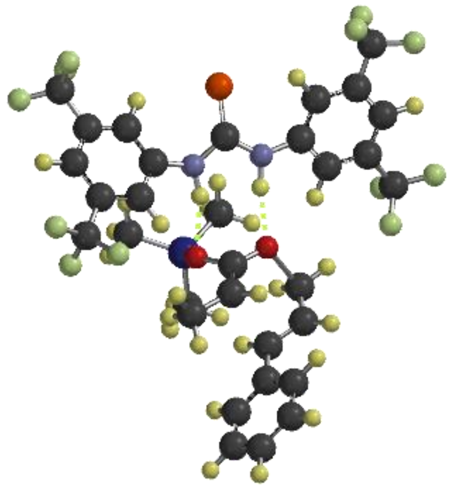

Method: ωB97X-D
Basis set: 6-31G*
Energy: -3382.466567 hartrees

Imaginary vibration: -446 cm^{-1} (Uncorrected); -420 cm^{-1} (Corrected); Intensity 28.45

Zero Point Energy : 1447.31 kJ/mol (ZPE)
Temperature Correction : 111.58 kJ/mol (vibration + gas law + rotation + translation)
Enthalpy Correction : 1558.89 kJ/mol (ZPE + temperature correction)
Enthalpy : -3381.872818 au (Electronic Energy + Enthalpy Correction)
Entropy : 1318.71 J/mol•K
Gibbs Energy : -3382.022569 au (Enthalpy - T*Entropy)
Cv : 815.73 J/mol•K

Energy (ωB97X-D/6-31G*//M06-2X/6-311+G**): -3383.08112 au
[E(TS-Z-enol): -1024.64378 au; E(**C12**): -2358. 40001 au)

Coordinates:

C	0.150436	-1.107572	0.052682	H	-0.403918	-3.281922	2.902621
C	0.953432	-1.673614	2.537597	H	1.931834	-1.413130	2.148898
C	0.441069	-2.947928	2.307693	H	1.689506	-3.313734	0.637213
C	0.779053	-3.619816	1.149379	O	1.079907	-0.554628	-0.787941
H	0.646089	-1.121160	3.417721	Si	2.651887	0.053054	-0.564649

C	3.815719	-1.378751	-0.212019
C	3.022401	0.823659	-2.225001
C	2.700122	1.330270	0.805451
C	0.242636	-4.920707	0.730340
C	-0.715080	-7.413573	-0.116972
C	0.998750	-5.721733	-0.132915
C	-1.012913	-5.382010	1.148811
C	-1.485669	-6.617496	0.729270
C	0.527922	-6.962156	-0.548982
H	2.345846	1.660605	-2.429417
H	2.901159	0.093436	-3.031992
H	3.691981	-2.171220	-0.958741
H	4.048639	1.205511	-2.264242
H	4.856154	-1.036753	-0.264994
H	1.993956	2.145239	0.612547
H	2.466315	0.909100	1.787789
H	3.701543	1.773120	0.862706
H	1.967640	-5.367920	-0.475499
H	1.130926	-7.574116	-1.212161
H	3.673496	-1.821189	0.779886
H	-1.632795	-4.762386	1.789992
H	-2.461960	-6.959857	1.057733
H	-1.088182	-8.378905	-0.444067
O	0.007427	-0.598179	1.223178
C	-0.525667	-2.222383	-0.383466
C	-0.363599	-2.821513	-1.745954
H	-0.524388	-3.903988	-1.709000
H	0.634790	-2.629436	-2.148104
H	-1.089885	-2.414078	-2.459414
H	-1.388653	-2.507406	0.205950
C	-1.022155	2.802880	-0.113422
S	-1.793229	4.254360	-0.393615
N	-0.844678	2.204261	1.101608
N	-0.463298	2.033642	-1.106188
C	-1.104012	2.635031	2.411352

C	-1.514179	3.227366	5.128562
C	-1.929863	3.699348	2.779947
C	-0.492177	1.875187	3.413083
C	-0.704164	2.163561	4.751579
C	-2.113845	3.983178	4.129495
C	-0.635514	2.130505	-2.501019
C	-0.894518	2.133664	-5.286161
C	-0.849043	0.937901	-3.190934
C	-0.528116	3.327768	-3.207534
C	-0.671563	3.316326	-4.590307
C	-0.972276	0.943839	-4.573814
C	-0.625126	4.621999	-5.340204
C	-2.953357	5.175959	4.509520
C	-0.001075	1.326897	5.783167
C	-1.144537	-0.364661	-5.294131
F	-0.201136	4.451371	-6.604262
F	-1.713289	-0.206086	-6.497784
F	-1.914274	-1.219443	-4.593195
F	-1.837724	5.193731	-5.403588
F	0.037896	-0.977333	-5.487988
F	0.199875	5.502075	-4.752063
F	-3.945267	5.389227	3.633561
F	-2.212608	6.296346	4.553253
F	-3.509349	5.023136	5.723833
F	-0.070462	0.010540	5.480970
F	-0.523162	1.480138	7.006146
F	1.306082	1.629992	5.860086
H	-2.425962	4.288091	2.022560
H	0.147025	1.046955	3.129111
H	-1.684006	3.453514	6.173530
H	-0.045120	1.150249	-0.833028
H	-0.446586	1.263310	1.090202
H	-0.337851	4.252781	-2.681966
H	-1.000658	2.140143	-6.363907
H	-0.924619	0.007519	-2.638382

(*E*)-Silyl ketene acetal + Ph-thiourea

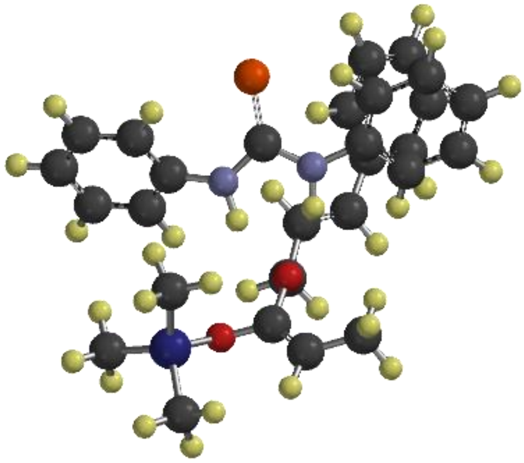

Method: ωB97X-D
Basis set: 6-31G*
Energy: -2034.684092 hartrees

Zero Point Energy : 1405.33 kJ/mol (ZPE)
Temperature Correction : 80.72 kJ/mol (vibration + gas law + rotation + translation)
Enthalpy Correction : 1486.05 kJ/mol (ZPE + temperature correction)
Enthalpy : -2034.118085 au (Electronic Energy + Enthalpy Correction)
Entropy : 1020.04 J/mol•K
Gibbs Energy : -2034.233920 au (Enthalpy - T*Entropy)
Cv : 593.35 J/mol•K

Energy (ωB97X-D/6-31G*//M06-2X/6-311+G**): -2034.91230 au
[E(E-enol): -1024.67697 au; E(Ph-thiourea): -1010.20040 au]

Coordinates:

C	1.203842	0.271462	-2.480163
C	1.627771	-1.284211	-0.722448
C	0.964102	-1.780121	0.518654
C	0.515732	-3.031343	0.651498
O	2.281576	1.036390	-2.211083
Si	2.422538	2.703171	-2.548862
C	3.969601	3.212756	-1.636641
C	2.628708	2.964789	-4.396596
C	0.886745	3.572373	-1.924334
C	-0.247539	-3.564965	1.790256
C	-1.734713	-4.642010	3.905264
C	-0.287528	-4.947248	2.003918
C	-0.978926	-2.729992	2.644047
C	-1.715039	-3.265343	3.691977
C	-1.018539	-5.483070	3.058192
O	0.715704	-0.356354	-1.359243
C	0.614160	0.106160	-3.664752
C	-0.572270	-0.775208	-3.916805
C	-1.318845	0.874635	1.194071
S	-2.007626	1.103576	2.701774
N	-0.157405	1.473649	0.781182
N	-1.802981	0.041805	0.224434
C	0.715883	2.299643	1.523406
C	2.560925	3.943284	2.828105
C	0.273454	3.413749	2.238022
C	2.083242	2.023585	1.450523
C	3.000910	2.843954	2.097975
C	1.197674	4.220822	2.891720
C	-2.898766	-0.851045	0.271160
C	-4.994745	-2.695537	0.206208

C	-2.729679	-2.091672	-0.349068
C	-4.129615	-0.524118	0.840101
C	-5.163994	-1.453377	0.810628
C	-3.772648	-3.007661	-0.381632
H	-0.784707	3.637394	2.280736
H	2.427256	1.165566	0.878643
H	3.274068	4.580776	3.341182
H	-1.163095	-0.147816	-0.541572
H	0.268945	1.071658	-0.049384
H	-4.269492	0.442165	1.305205
H	-5.809416	-3.413068	0.191548
H	-1.764205	-2.343142	-0.779670
H	-6.116811	-1.195204	1.263011
H	-3.621980	-3.972592	-0.856156
H	0.845285	5.082141	3.450975
H	4.060578	2.613713	2.037289
H	3.831066	3.118149	-0.555253
H	4.217468	4.258536	-1.851880
H	-1.034561	-6.557682	3.213962
H	-0.356376	-1.507218	-4.703498
H	1.831142	-2.099507	-1.426806
H	0.712694	-3.739778	-0.155545
H	0.265932	-5.606610	1.339261
H	-2.315572	-5.058943	4.722400
H	-2.285596	-2.599617	4.332104
H	-1.005190	-1.656984	2.480009
H	0.822603	-1.054165	1.317098
H	4.824946	2.595610	-1.932271
H	1.686746	2.839188	-4.940626
H	1.025612	0.656682	-4.504416
H	-1.437131	-0.189664	-4.251239
H	-0.866417	-1.321413	-3.017094
H	0.838672	4.592594	-2.322298
H	3.363453	2.269849	-4.817449
H	2.984949	3.983500	-4.590817
H	0.875415	3.639040	-0.831828
H	-0.022668	3.051402	-2.244640
H	2.564670	-0.757089	-0.512468

TS-(*E*)-Ph-thiourea-catalyzed

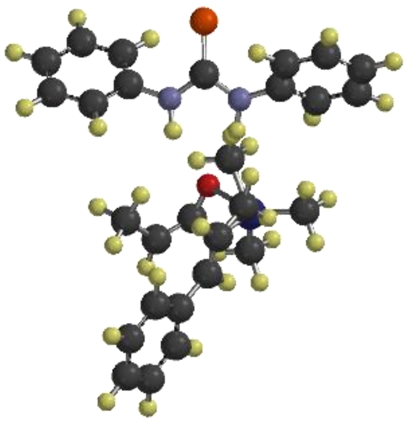

Method: ωB97X-D
Basis set: 6-31G*
Energy: -2034.645454 hartrees

Imaginary vibration: -435 cm^{-1} (Uncorrected); -410 cm^{-1} (Corrected); Intensity 31.39

Zero Point Energy : 1398.15 kJ/mol (ZPE)
Temperature Correction : 81.60 kJ/mol (vibration + gas law + rotation + translation)
Enthalpy Correction : 1479.75 kJ/mol (ZPE + temperature correction)
Enthalpy : -2034.081849 au (Electronic Energy + Enthalpy Correction)
Entropy : 967.88 J/mol•K
Gibbs Energy : -2034.191761 au (Enthalpy - T*Entropy)

Cv : 600.21 J/mol•K

Energy (ωB97X-D/6-31G*//M06-2X/6-311+G**): -2034.87339 au
[E(TS-E-enol): -1024.64863 au; E(Ph-thiourea): -1010.20123 au)]

Coordinates:

C	0.527061	-0.320423	-1.204510
C	1.154097	-1.297327	1.156729
C	0.891822	-2.543251	0.580256
C	1.420755	-2.823361	-0.663295
H	0.680027	-1.019400	2.092409
H	0.086869	-3.147594	0.987617
H	2.109034	-0.819566	0.964721
H	2.249199	-2.202814	-0.999660
O	1.667119	0.202738	-1.719689
Si	2.345860	1.718076	-1.363323
C	3.503560	1.564550	0.105629
C	3.324913	2.111192	-2.910051
C	1.007305	2.993870	-1.068191
C	1.207114	-4.038310	-1.457968
C	0.891288	-6.329211	-3.045083
C	1.949757	-4.206223	-2.634656
C	0.296274	-5.038439	-1.094476
C	0.143017	-6.174140	-1.881208
C	1.795940	-5.339218	-3.421434
H	2.664138	2.181569	-3.780490
H	4.065205	1.330481	-3.115508
H	4.253136	0.783809	-0.066193
H	3.858886	3.063089	-2.813504
H	4.041003	2.506980	0.265776
H	0.285009	3.002681	-1.891815
H	0.451005	2.824759	-0.141706
H	1.453884	3.993596	-1.010012
H	2.651839	-3.431349	-2.931770
H	2.382499	-5.451318	-4.327857
H	2.971999	1.326757	1.032759
H	-0.291779	-4.936890	-0.188107
H	-0.562473	-6.943012	-1.582304
H	0.770724	-7.218228	-3.655994
O	0.217731	-0.040077	0.017785
C	-0.101221	-1.303572	-1.926917
C	-1.415055	-1.899819	-1.539827
H	0.258231	-1.472837	-2.937316
H	-1.436689	-2.974249	-1.757938
H	-1.613946	-1.752846	-0.474735
H	-2.234143	-1.437632	-2.105548
C	-1.839669	2.022673	1.929050
S	-2.814712	2.923204	2.943459
N	-2.200107	1.570446	0.690338
N	-0.560345	1.640840	2.228100
C	-3.390428	1.850905	-0.019601
C	-5.675144	2.328368	-1.550214
C	-3.851817	3.156056	-0.195303
C	-4.070836	0.789788	-0.616113
C	-5.205201	1.029819	-1.385060
C	-4.995442	3.385807	-0.949548
C	0.129122	1.793339	3.452937
C	1.633323	1.965903	5.796540
C	1.426697	2.302529	3.418732
C	-0.419582	1.372430	4.665204
C	0.331352	1.470364	5.829885
C	2.179913	2.379029	4.586231
H	-3.318653	3.978162	0.265627
H	-3.714881	-0.224713	-0.461854
H	-6.565825	2.517252	-2.141273
H	-0.023722	1.238737	1.465300
H	-1.594199	0.863290	0.281002
H	-1.430934	0.984825	4.686940
H	2.215085	2.033154	6.710447
H	1.830538	2.660316	2.475872
H	-0.103264	1.148084	6.771119
H	3.189345	2.776848	4.548281
H	-5.353201	4.403231	-1.074936
H	-5.728416	0.196271	-1.843756

(*E*)-Silyl ketene acetal + squaramide

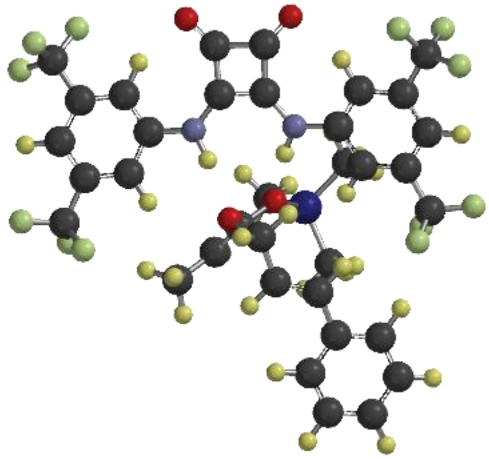

Method: ωB97X-D
Basis set: 6-31G*
Energy: -3248.981166 hartrees

Zero Point Energy : 1514.98 kJ/mol (ZPE)
Temperature Correction : 116.52 kJ/mol (vibration + gas law + rotation + translation)
Enthalpy Correction : 1631.50 kJ/mol (ZPE + temperature correction)
Enthalpy : -3248.359763 au (Electronic Energy + Enthalpy Correction)
Entropy : 1383.07 J/mol•K
Gibbs Energy : -3248.516823 au (Enthalpy - T*Entropy)
Cv : 852.91 J/mol•K

Energy (ωB97X-D/6-31G*//M06-2X/6-311+G**): -3249.64000 au
[E(E-enol): -1024.67824 au; E(squaramide): -2224.91638 au]

Coordinates:

C	-1.335377	-0.341787	0.119867	C	-1.877574	-3.651286	-3.732159
C	0.278883	-2.101318	-0.064919	C	-2.539499	-3.959353	-4.912915
C	-0.479386	-2.585081	-1.255677	C	-0.982911	-2.598588	-6.146221
C	-0.058103	-2.402945	-2.510055	H	1.081939	2.409679	-2.596676
H	0.564433	-2.917330	0.604413	H	0.884698	3.106642	-0.977380
H	-1.425748	-3.078166	-1.043734	H	-2.392581	2.977109	0.173898
H	1.188151	-1.567437	-0.361282	H	0.048598	3.820811	-2.358337
H	0.890391	-1.891218	-2.669223	H	-2.516928	3.820229	-1.374416
O	-0.597917	0.653366	-0.436608	H	-0.768225	0.368218	-3.614330
Si	-1.133404	1.791119	-1.617109	H	-2.333154	0.011373	-2.878607
C	-2.524945	2.830578	-0.903598	H	-2.129798	1.469133	-3.866420
C	0.365040	2.870924	-1.912668	H	0.553792	-1.646077	-4.986368
C	-1.640041	0.820504	-3.131420	H	-0.628056	-2.187275	-7.086265
C	-0.761782	-2.803912	-3.738468	H	-3.518242	2.401152	-1.066208
C	-2.097648	-3.430081	-6.124260	H	-2.226515	-4.084694	-2.799575
C	-0.317618	-2.293270	-4.963903	H	-3.401803	-4.618766	-4.889797

H	-2.617200	-3.671630	-7.046378	F	7.568497	1.190597	-1.832183
O	-0.539588	-1.246257	0.771999	F	3.956829	0.241926	-4.968444
C	-2.663381	-0.450096	0.141001	F	1.910011	0.466974	-4.293210
C	-3.419545	-1.601533	0.730369	F	6.777099	3.108750	-1.224497
H	-3.248322	0.348820	-0.299937	F	2.954323	-1.394730	-3.972562
H	-4.068945	-2.061971	-0.024073	F	7.135539	1.573348	0.255206
H	-2.748470	-2.372777	1.115914	F	-1.206850	3.074870	7.148956
H	-4.064403	-1.275204	1.552592	F	-1.076200	1.278851	8.345600
N	0.196638	0.460534	3.040245	F	-3.011726	2.094281	7.834877
N	2.114272	0.944135	0.674898	F	-4.890547	-1.197157	3.920772
C	-0.770327	0.367741	4.049599	F	-4.371852	-2.188423	5.768712
C	-2.793100	0.082352	5.961406	F	-3.438391	-2.799158	3.911825
C	-0.779374	1.189005	5.177679	H	0.002664	1.924824	5.337259
C	-1.769702	-0.594124	3.880437	H	-1.754147	-1.238170	3.007327
C	-2.767992	-0.730417	4.834083	H	-3.573331	-0.026223	6.704838
C	-1.792559	1.034145	6.117098	H	1.167229	0.732918	0.359206
C	3.083241	0.931592	-0.330908	H	0.053282	-0.174297	2.254640
C	4.911285	0.838495	-2.458017	H	4.732060	1.716336	0.831028
C	2.685533	0.480152	-1.591167	H	5.620028	0.805130	-3.275709
C	4.406351	1.336395	-0.133232	H	1.655271	0.178402	-1.752764
C	5.298523	1.280531	-1.196794	C	1.328471	1.195030	3.007765
C	3.592707	0.445651	-2.640906	C	2.228684	1.362505	1.961961
C	6.704760	1.788016	-0.993644	C	2.147984	2.025960	3.933831
C	-1.770489	1.879410	7.365697	C	3.172234	2.148527	2.795575
C	-3.865966	-1.735719	4.613467	O	2.045605	2.458788	5.053500
C	3.116460	-0.055346	-3.974463	O	4.268311	2.636225	2.670058

TS-(*E*)-Ph-squaramide-catalyzed

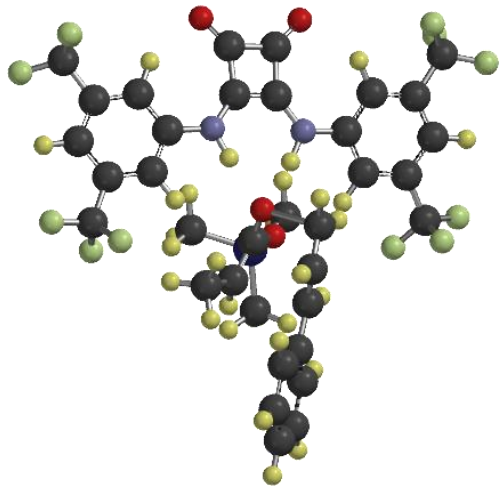

Method: ωB97X-D
Basis set: 6-31G*
Energy: -3248.945799 hartrees

Imaginary vibration: -417 cm^{-1} (Uncorrected); -393 cm^{-1} (Corrected); Intensity 37.36

Zero Point Energy : 1507.02 kJ/mol (ZPE)
Temperature Correction : 117.46 kJ/mol (vibration + gas law + rotation + translation)
Enthalpy Correction : 1624.48 kJ/mol (ZPE + temperature correction)
Enthalpy : -3248.327067 au (Electronic Energy + Enthalpy Correction)
Entropy : 1340.16 J/mol•K
Gibbs Energy : -3248.479255 au (Enthalpy - T*Entropy)
Cv : 859.97 J/mol•K

Energy (ωB97X-D/6-31G*//M06-2X/6-311+G**): -3249.60586 au
[E(TS-E-enol): -1024.64290 au; E(squaramide): -2224.91621 au]
Coordinates:

C	-0.243101	-0.843765	-0.492015
C	1.326652	-1.626672	1.491546
C	0.977522	-2.916166	1.099586
C	1.043456	-3.256218	-0.239123
H	1.207385	-1.314872	2.522883
H	0.435140	-3.543892	1.799946
H	2.075652	-1.081169	0.926267
H	1.648519	-2.620578	-0.883950
O	0.608037	-0.163832	-1.311174
Si	0.275974	0.415623	-2.883672
C	-1.451113	1.138735	-2.877056
C	1.587503	1.710989	-3.169126
C	0.439155	-0.993751	-4.115392
C	0.650198	-4.539720	-0.828601
C	-0.050704	-6.969783	-2.040645
C	0.994384	-4.803520	-2.161466
C	-0.060567	-5.515436	-0.115731
C	-0.406012	-6.719108	-0.717604
C	0.651010	-6.007066	-2.762530
H	2.593819	1.289972	-3.083841
H	1.501692	2.526310	-2.442526
H	-1.529027	1.960762	-2.156991
H	1.488374	2.142036	-4.172452
H	-1.706594	1.536735	-3.865793
H	1.283060	-1.641991	-3.852557
H	-0.462127	-1.613902	-4.169456
H	0.628053	-0.601433	-5.121292
H	1.541528	-4.053391	-2.727213
H	0.932301	-6.196423	-3.793718
H	-2.206268	0.390240	-2.614455
H	-0.348048	-5.337988	0.915609
H	-0.954880	-7.464832	-0.150914
H	-0.319532	-7.912538	-2.506983
O	-0.139222	-0.532467	0.753910
C	-0.952716	-1.932883	-0.938311
C	-2.007715	-2.624473	-0.137172
H	-0.925911	-2.149824	-2.002257
H	-1.994864	-3.703107	-0.330588
H	-1.865279	-2.461123	0.934276
H	-3.006613	-2.265707	-0.409527
N	-1.550184	1.706319	1.833112
N	1.423917	2.017684	1.066133
C	-2.943989	1.637299	1.890861
C	-5.729851	1.344298	1.871070
C	-3.756910	2.703678	2.279899
C	-3.525519	0.427037	1.508131
C	-4.905788	0.289798	1.497959
C	-5.137534	2.540375	2.262287
C	2.800457	2.117756	0.838208
C	5.557568	2.155345	0.341876
C	3.365844	1.225597	-0.076242
C	3.615305	3.038691	1.500151
C	4.980443	3.045224	1.241724
C	4.733014	1.249984	-0.314975
C	5.844781	4.075270	1.926006
C	-5.999056	3.690219	2.721211
C	-5.482557	-1.012066	1.013928
C	5.322601	0.234254	-1.254745
F	7.106872	3.636879	2.075050
F	6.478414	0.649960	-1.787841
F	4.485704	-0.049212	-2.275997
F	5.901865	5.212464	1.215634
F	5.565379	-0.931198	-0.629183
F	5.377991	4.385661	3.142977
F	-5.530383	4.864816	2.277730
F	-6.043544	3.759248	4.061595
F	-7.264875	3.568198	2.286851
F	-5.303843	-1.158624	-0.316134
F	-6.795975	-1.112009	1.250444
F	-4.882180	-2.065976	1.596769

C	-0.738105	2.772326	2.009434	H	6.624676	2.170615	0.157251
C	0.613098	2.894314	1.703027	H	2.723982	0.535725	-0.615530
C	-0.808360	4.146111	2.571544	H	0.978168	1.170558	0.713057
C	0.684683	4.275597	2.248082	H	-1.094242	0.850810	1.512658
O	-1.655248	4.863538	3.044630	H	-2.890375	-0.400677	1.208649
O	1.531320	5.121704	2.396974	H	-6.806967	1.239844	1.853333
H	3.190263	3.760487	2.189192	H	-3.319970	3.648305	2.593318

Kinetic measurements

For kinetic measurements, ^{1}H NMR spectra were recorded every 10 minutes for 15 hours. The relative concentrations of initial compound **2c**, product **3c** and unidentified by-product were determined through integration of selected signals (5.96 ppm, 6.31 ppm and 7.0 ppm, respectively) (Figure S6). Corresponding rate constants for initial compound consumption and product(s) formation were obtained by monoexponential fitting of the experimental data (equation S1).

$$y = A_1\, e^{-x/t_1} + y_0 \qquad \text{(S1)}$$

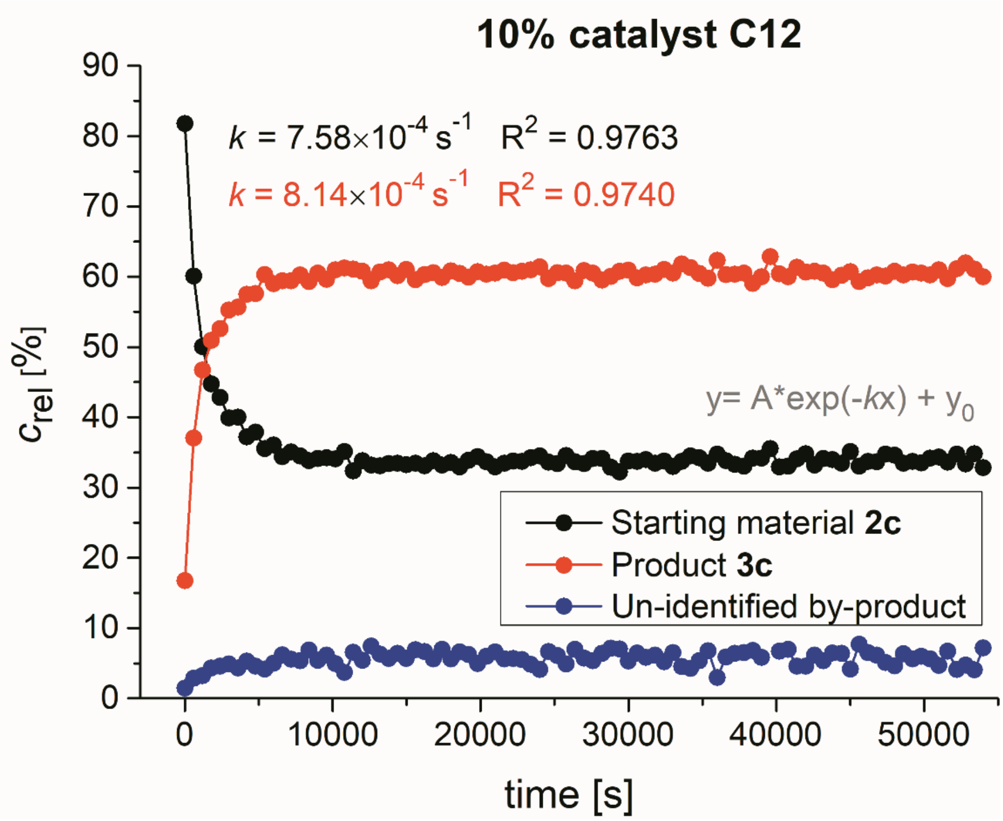

Figure S1.

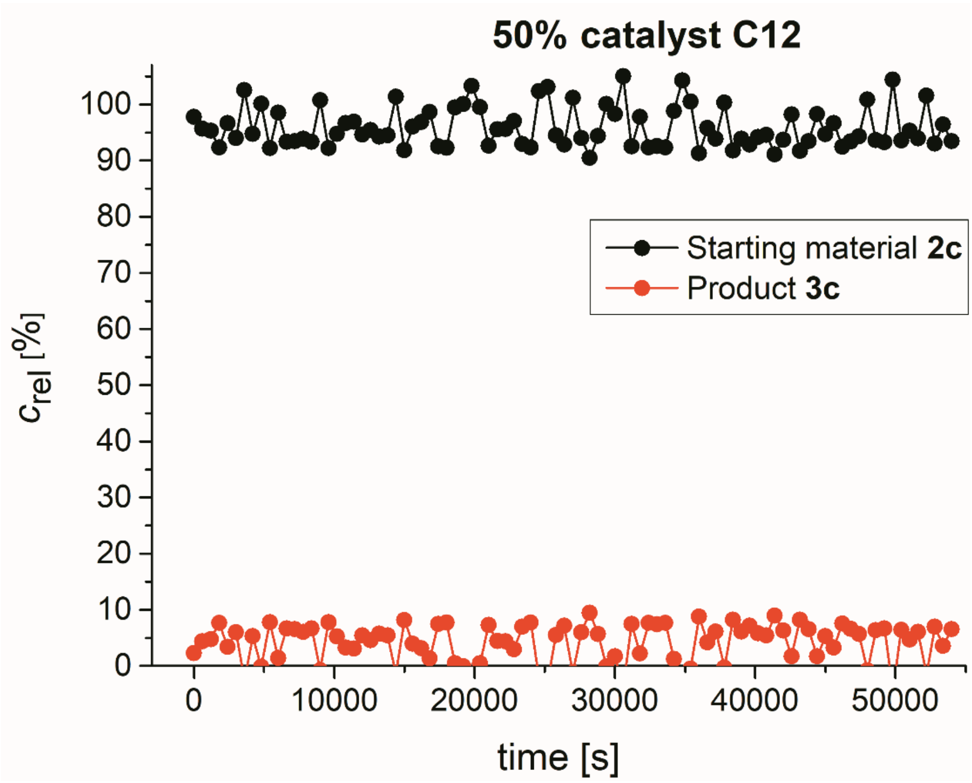

Figure S2.

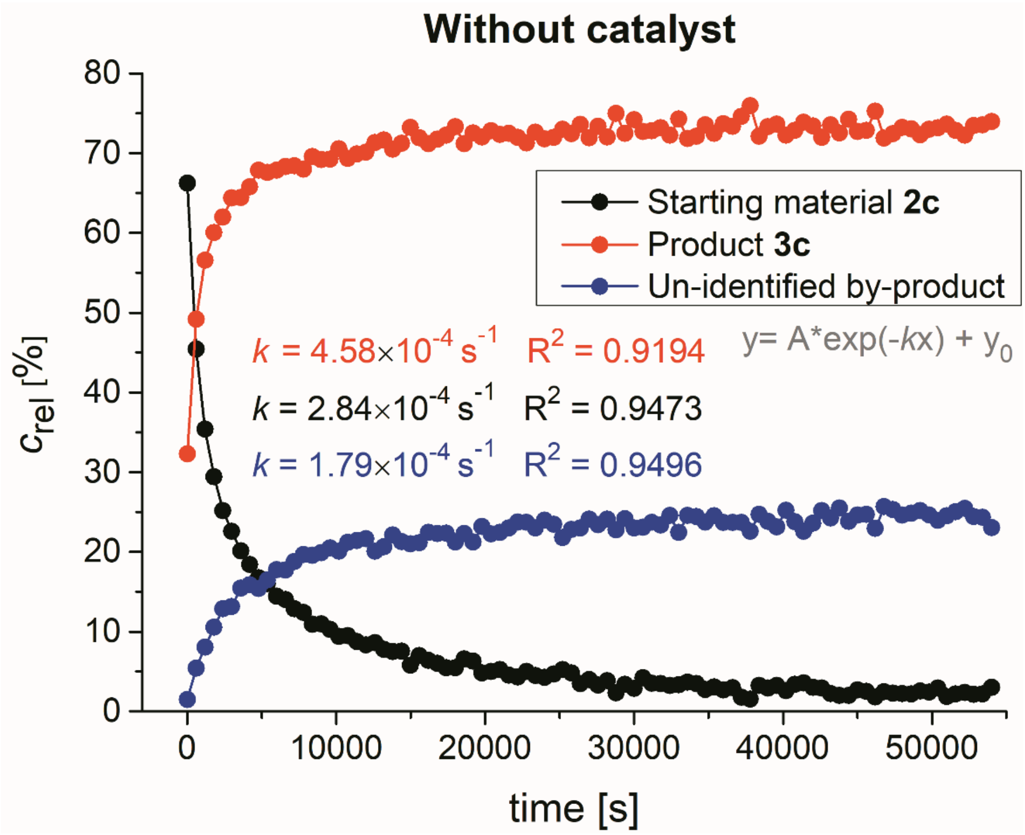

Figure S3.

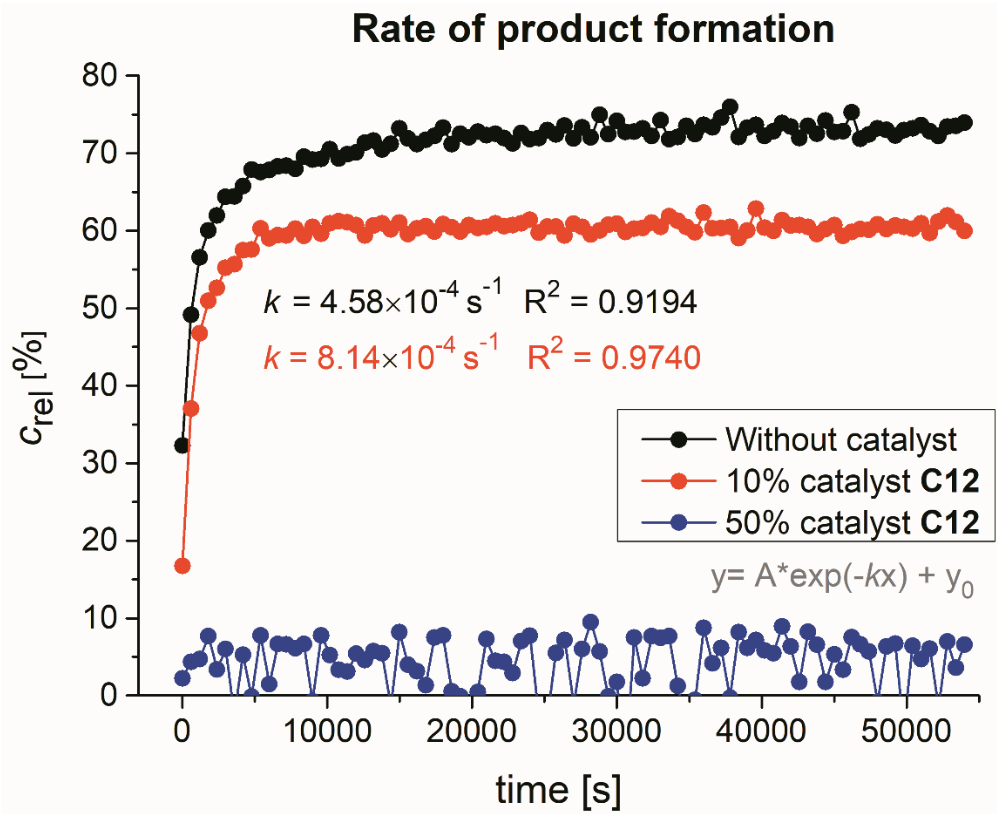

Figure S4.

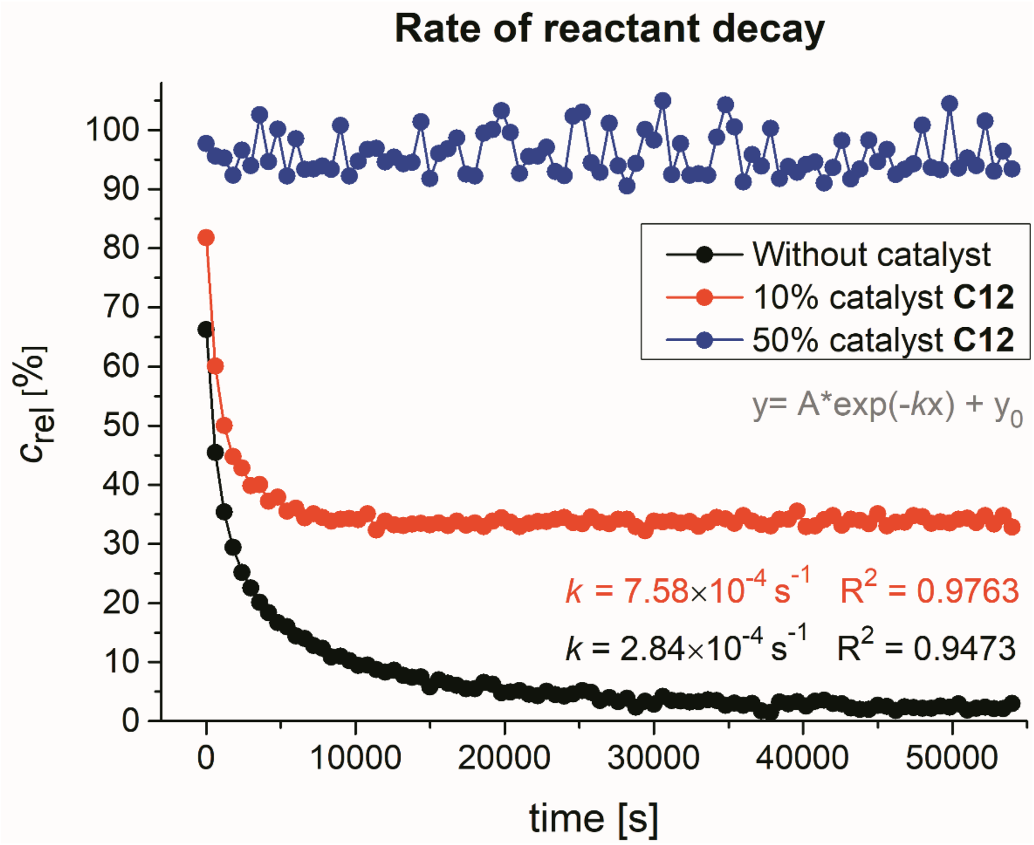

Figure S5.

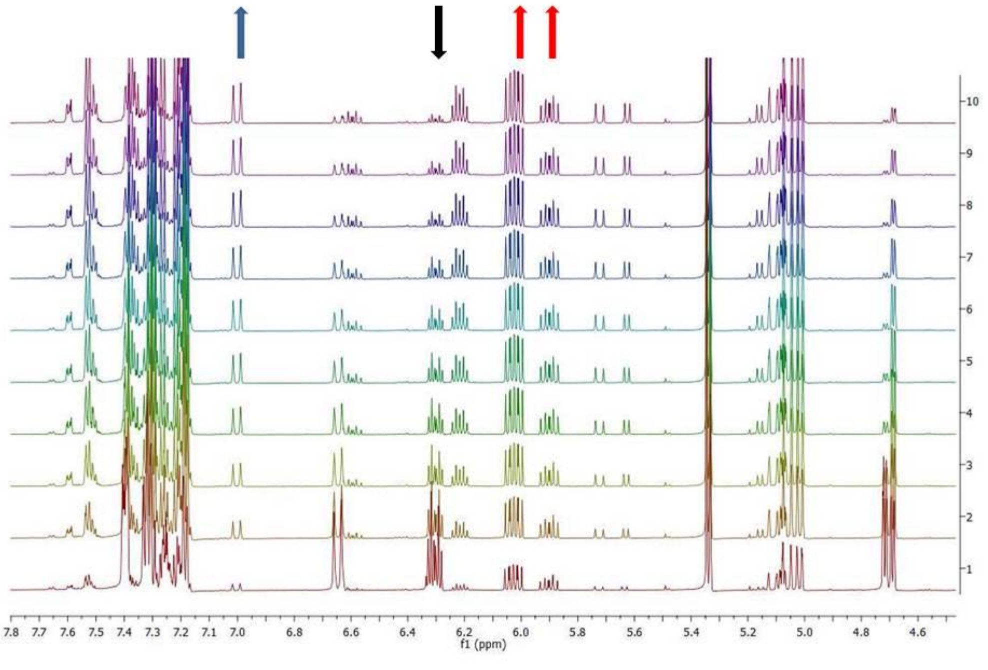

Figure S6
